# Beyond static snapshots: gated recurrent units and graph attention networks for smarter multi-agent traffic control

**DOI:** 10.3389/frai.2026.1891950

**Published:** 2026-09-18

**Authors:** Mohita, Sidak Deep Singh, D. Subitha, J. C. Kavitha

**Affiliations:** School of Computer Science and Engineering (SCOPE), Vellore Institute of Technology (VIT), Chennai, India

**Keywords:** gated recurrent units, graph attention networks, intelligent transportation systems, multi-agent reinforcement learning, multi-objective optimization, robust control, spatio-temporal modeling, traffic signal control

## Abstract

Existing multi-agent reinforcement learning (MARL) approaches for traffic signal control often fail to capture two critical characteristics of real-world urban traffic: the dynamic spatio-temporal propagation of congestion across intersections and the frequent imperfection of sensor data. To address these fundamental gaps, this paper introduces hybrid coordinated spatio-temporal graph reinforcement learning (HC-STGRL), a novel framework that integrates three complementary mechanisms-gated recurrent unit (GRU), graph attention network (GAT), dueling double deep Q-Network (3DQN). Each intersection is equipped with a GRU to retain and update short-term memory of evolving traffic patterns, while a GAT enables selective and adaptive information sharing with neighboring intersections, allowing agents to weigh the relevance of spatio-temporal dependencies from different sources. The final phase decisions of the signal are derived using 3DQN. The proposed system is extensively evaluated using SUMO simulations on two synthetic grid networks and two real-world traffic networks. Experimental results demonstrate that HC-STGRL consistently reduces average trip times and delays compared to state-of-the-art MARL baselines, achieving up to a 11.3% reduction in travel time and a 14.9% reduction in waiting time relative to the strongest baseline, CoLight, on real-world road networks. Furthermore, the framework exhibits notable robustness, maintaining efficient operation even when 20% of sensors malfunction, a condition that significantly degrades the performance of existing methods: HC-STGRL incurs only a 25.4% performance degradation under 20% sensor failure, compared with 44.7% for CoLight. Importantly, HC-STGRL incorporates built-in mechanisms to prevent major arterial roads from starving adjacent intersections, thereby overcoming a well-documented limitation of conventional throughput-maximizing control strategies.

## 1 Introduction

Urban traffic congestion has emerged as a critical challenge in the current era, where fuel crises amplify economic instability, environmental degradation, industrial disruptions, and inconveniences of daily life. According to a recent study by the Texas A&M Transportation Institute in 2021, Americans stuck in traffic lost 4.3 billion hours of their lives and burned an additional 2.9 billion gallons of gasoline, which is equivalent to $190 billion in total costs (Schrank et al., 2021). This disruption significantly affects individuals' lives and serves as a major contributor to urban air pollution. Urban intersections experience trip delays between 40 and 55%, varying with respect to time and location (Schrank et al., 2021).

Major Indian metropolitan cities continue to experience severe traffic congestion that significantly affects the daily lives of commuters. Bengaluru, in particular, frequently suffers from bottlenecks, long commute times, and increased waiting times. The main reason behind all these problems is rapid urbanization and exponential growth of vehicles. Similarly, Chennai is another metro city that faces high congestion during peak hours and in heavy rain fall conditions, leading to delays, economic losses, and increased air pollution. These challenges highlight the urgent need for intelligent transportation systems capable of providing real-time traffic forecasting and adaptive traffic control to improve urban mobility and reduce the socio-economic impact of congestion.

Intelligent Transportation Systems (ITS) play a vital role in mitigating these issues through efficient traffic forecasting and real-time traffic control (Papageorgiou et al., 2003; Xiao et al., 2026). Traffic forecasting involves predicting future traffic conditions, congestion levels, and vehicle flow using real-time and historical data combined with artificial intelligence and deep learning techniques (Yu et al., 2018; Xing et al., 2026). Based on these predictions, intelligent traffic control systems can dynamically regulate traffic signals, optimize routing, and manage road usage to reduce congestion, fuel consumption, travel delays, and environmental pollution (Papageorgiou et al., 2003). Together, traffic forecasting and traffic control form the predictive and operational backbone of modern ITS, enabling smarter and more adaptive transportation management.

Traditional traffic signal control is based on fixed timers based on historical averages (Lieberman, 2000). These traffic lights operate in predefined patterns throughout the day, without adapting to real-time street conditions. More advanced systems like SCOOT and SCATS, deployed in various cities during the 1980s, (Hunt et al., 1981; Lowrie, 1990) use embedded induction loops on the pavement to detect vehicles and adjust signal timings accordingly, such as extending the duration of green light when queues form. However, these systems remain fundamentally reactive as they respond to the current states rather than anticipating future changes.

Recent advances in deep learning techniques have been instrumental in the growth of ITS by modeling complex traffic characteristics using advanced models such as recurrent neural networks (RNNs), graph neural networks (GNNs), and transformer-based architectures (Yu et al., 2018; Wu et al., 2023; Jiang et al., 2024). These models help to achieve state-of-the-art performance in traffic prediction and congestion analysis (Xiao et al., 2025, 2026). These models make use of the real-time data collected from different types of sensors, GPS devices and cameras that provide required information for accurate real-time traffic forecasting (Yu et al., 2018).

Furthermore, in dynamic traffic environments, RL has emerged as an effective approach to adaptive traffic control and intelligent decision-making (Mnih et al., 2015; Abdulhai et al., 2003). Using a MARL framework, each traffic junction is treated as an independent intelligent agent that observes local traffic conditions through continuous interaction with the environment and learns an optimal traffic control signal. RL offers a fundamentally different approach. An RL agent learns effective solutions through experience rather than following predetermined rules (Mnih et al., 2015). By testing various signal timings and observing their impact on queue lengths and delays, the agent develops a state-to-action mapping that creates effective traffic control strategies (Mnih et al., 2015; van Hasselt et al., 2016).

Generalizing reinforcement learning (RL) algorithms to situations that require more than one intersection leads to the development of MARL, where each intersection is considered a learning agent that interacts with other intersections (Abdulhai et al., 2003; Busoniu et al., 2008). While this enables network-wide traffic optimization, the early approach to multi-agent RL has been problematic due to the non-stationarity issue since early approaches treated nearby agents as part of the environment; hence, any changes made at one intersection to its policy would affect the environment of others, leading to poor coordination and performance (El-Tantawy et al., 2013; Chu et al., 2020).

Recent graph-based MARL frameworks such as CoLight (Wei et al., 2019) and MPLight (Chen et al., 2020), graph-based coordination is improved through the communication between adjacent intersections using graph attention. Recent surveys (Xiao et al., 2025, 2026) suggest that graph-based spatial coordination has become the dominant paradigm in network-wide traffic light control. Nevertheless, such paradigms only consider the interactions in space on an individual time step without explicitly modeling the dynamics in time. Although recent transformer-based controllers (Wu et al., 2023; Jiang et al., 2024) introduce sequence modeling, they generally incur substantially higher computational and memory costs, motivating the lightweight recurrent design adopted in this work.

Another critical issue is sensor reliability. Academic research often assumes perfect sensor accuracy-that agents always receive complete and reliable information (Tan et al., 2020; Zhan, 2026). In reality, sensors often fail. Cameras become dirty, lose focus, or get blocked by construction. Power supplies fluctuate and communication links drop packets or lose connectivity entirely. Field studies report that such sensor failures, together with missing-data, are common in deployed traffic networks and can substantially degrade the accuracy of the data (Zhan, 2026). Most MARL systems require near-perfect data to operate correctly and degrade dramatically when this assumption is violated (Tan et al., 2020; Zhan, 2026). A failed sensor creates a blind spot, leaving the agent unable to access critical local information for policy implementation.

In summary, two research gaps remain inadequately addressed by existing graph-based MARL controllers: (i) the absence of explicit temporal memory, which prevents agents from distinguishing rapidly evolving traffic conditions from stable congestion patterns and therefore limits anticipatory traffic control; and (ii) insufficient robustness to imperfect or missing sensor observations, which can significantly degrade performance under realistic operating conditions.

To address these limitations, this paper proposes HC-STGRL, a hybrid coordinated spatio-temporal graph reinforcement learning framework integrating a Gated Recurrent Unit (GRU), a Graph Attention Network (GAT), and a Dueling Double Deep Q-Network (3DQN). The GRU models temporal traffic dynamics, the GAT enables adaptive information sharing among neighboring intersections, and the 3DQN learns an optimal traffic signal control policy from the resulting spatio-temporal representations.

Experimental evaluation in synthetic and real-world SUMO traffic networks demonstrates that HC-STGRL consistently outperforms existing MARL baselines in average travel time, average waiting time, and throughput across all evaluated networks (Table 1). Furthermore, HC-STGRL maintains robust performance under sensor failures (Table 2), while ablation studies confirm the contribution of each architectural component to overall performance improvements (Table 3).

**Table 1:** Performance across networks (ATT and AWT in seconds).

Model	Grid 4×4		Cologne8		Ingolstadt21		Manhattan	
	ATT	AWT	ATT	AWT	ATT	AWT	ATT	AWT
Fixed-time	205.07	132.79	114.39	65.15	287.71	184.89	421.35	267.42
MaxPressure	162.34	98.56	96.78	52.34	218.45	138.67	356.78	219.45
IDQN	158.67	95.34	92.45	49.78	205.67	132.45	345.89	208.67
MPLight	142.56	82.34	84.67	44.56	189.34	118.56	312.45	187.34
CoLight	138.45	78.67	81.23	42.34	178.56	108.67	298.67	176.45
**HC-STGRL**	**126.78**	**68.45**	**74.56**	**36.78**	**158.34**	**92.45**	**267.89**	**154.67**

Bold values indicate the best performance in each category.

**Table 2:** Robustness under sensor failure (Ingolstadt21—ATT in seconds).

Algorithm	0% fail	5% fail	10% fail	15% fail	20% fail
MaxPressure	218.45	245.67	278.34	312.56	354.78
MPLight	189.34	204.56	223.45	248.67	278.34
CoLight	178.56	191.23	208.45	231.67	258.34
**HC-STGRL**	**158.34**	**163.45**	**171.23**	**182.45**	**198.67**

Bold values indicate the best performance in each category.

**Table 3:** Ablation study results (Ingolstadt21).

Architecture	ATT	AWT	Queue Var.	Convergence
	(s)	(s)	(σ^2^)	episodes
Full HC-STGRL	158.34	92.45	2.34	850
Variant 1 (w/o GRU)	178.56	108.67	8.78	1,250
Variant 2 (w/o GAT)	198.45	128.34	12.45	1,800
Variant 3 (w/o Entropy)	162.78	95.67	15.67	950
Variant 4 (w/o 3DQN)	168.34	101.23	4.56	1,450

The key contributions of this paper, which address these gaps, are as follows:

1. **Temporal memory for anticipatory control**. Each intersection agent is equipped with a Gated Recurrent Unit (GRU) that maintains a short-term memory of traffic evolution, enabling the agent to evaluate not only current conditions but also their rate of change. The proposed framework enables proactive traffic signal control by taking advantage of upstream traffic conditions, allowing downstream intersections to anticipate approaching vehicle platoons and adjust signal phases before their arrival.
2. **Adaptive spatial coordination**. Multi-head Graph Attention Networks (GAT) enable intersections to exchange temporal memory states with neighbors and to weight information from different sources dynamically, so that a rapidly growing queue is prioritized over a stable one. These priorities are automatically learned from experience, enabling green-wave formation.
3. **Robustness to sensor failures**. When an intersection loses its local sensors, the attention mechanism dynamically upweights neighboring agents, allowing the affected agent to infer its own congestion state from neighbors' observations.
4. **Fairness through spatial entropy**. A spatial entropy-enhanced reward function is proposed to balance traffic queues across all approaches, preventing side-street starvation while maintaining efficient arterial traffic flow during peak periods.
5. **Comprehensive validation**. Evaluation extends beyond synthetic grids to complex real-world networks: Cologne's eight irregularly spaced intersections, Ingolstadt's 21-intersection arterial corridor, and a large-scale 14 × 14 Manhattan grid with 196 intersections. Ablation studies confirm that every component (GRU, GAT, entropy term, and dueling architecture) contributes positively to overall performance.

The remainder of this paper is organized as follows. Section 2 reviews related work, covering the evolution of multi-agent traffic control and recent directions such as transformer-based, robust, explainable, and edge-deployed reinforcement learning. Section 3 formalizes the theoretical foundations, including Markov decision process formulation, state and action design, and deep reinforcement learning preliminaries. Section 4 presents the proposed HC-STGRL approach in detail. Section 5 describes the experimental study, including the simulation environment, networks, baselines, metrics, and reproducibility settings. Section 6 reports and discusses the results. Section 7 states the limitations of the study, and Section 8 concludes and outlines the directions for future work.

## 2 Related work

In this section, we review the evolution of multi-agent traffic signal control from independent learning to graph-based coordination, discuss the limitations of existing approaches, and identify the specific research gaps that HC-STGRL addresses.

Traffic signal control has progressed through several stages, each addressing limitations of its predecessors. Early approaches relied on fixed-time control based on Webster's equation (Webster, 1958), which optimizes cycle lengths using historical averages but cannot adapt to real-time fluctuations. Systems such as SCOOT and SCATS (Hunt et al., 1981; Lowrie, 1990) introduced reactive adjustments using inductive loop data, yet remain fundamentally limited by their inability to anticipate future traffic states.

Reinforcement learning (RL) emerged as a paradigm shift, enabling agents to learn optimal policies through interaction. Deep Q-Networks (DQN) (Mnih et al., 2015) successfully approximated *Q*-values for continuous state spaces. However, applying RL to traffic networks faces a fundamental challenge: the non-stationarity problem in multi-agent settings. When agents learn independently, as in Independent DQN (IDQN) (Arel et al., 2010), the environment for each agent changes as others update their policies, preventing convergence and causing unstable coordination (Equation 1):


$$\begin{array}{l} P\left(s_{t+1}|s_{t},a_{t}^{1},\ldots ,a_{t}^{N},\pi_{t}^{1},\ldots ,\pi_{t}^{N}\right)\neq P\left(s_{t+1}|s_{t},a_{t}^{1},\ldots ,a_{t}^{N}\right) \end{array}$$ (1)

This instability manifests itself as congestion displacement: an intersection improves its own throughput by pushing congestion downstream rather than reducing the network-wide delay (Chu et al., 2020).

To overcome the above issue, parameter sharing has emerged as a mitigation strategy, where a single network learns from collective experiences across all agents (Zang et al., 2020). The loss function aggregates experiences (Equation 2):


$$\begin{array}{l} L\left(\theta \right)=\underset{i\in N}{\sum }E_{\left(s,a,r,s^{\prime }\right)\sim D_{i}}\left[{\left(y_{i}-Q\left(s,a;\theta \right)\right)}^{2}\right] \end{array}$$ (2)

While this increases data efficiency and reduces non-stationarity, it assumes homogeneous intersection characteristics—an unrealistic simplification for real networks where corner intersections differ fundamentally from central ones.

Max-pressure theory (Varaiya, 2013) offers a theoretically grounded alternative. The parameter, pressure for phase *p* is defined as the difference between queues on the incoming and outgoing lanes. Controllers like PressLight (Chen et al., 2019) and MPLight (Chen et al., 2020) combine max-pressure state representations with RL. Although these techniques are effective under ideal conditions, these approaches suffer from two critical limitations: (i) they require precise lane-level sensor data, making them brittle under failures, and (ii) the pressure formulation lacks temporal context—it treats current queues as independent of their evolution.

Graph-based coordination represents the current state-of-the-art. CoLight (Wei et al., 2019) introduced Graph Attention Networks (GAT) to enable intersections to dynamically weight neighboring information (Equation 3):


$$\begin{array}{l} \alpha_{ij}=\frac{\exp\left(\text{LeakyReLU}\left({\text{a}}^{T}\left[\text{W}h_{i}||\text{W}h_{j}\right]\right)\right)}{\sum_{k\in N_{i}}\exp\left(\text{LeakyReLU}\left({\text{a}}^{T}\left[\text{W}h_{i}||\text{W}h_{k}\right]\right)\right)} \end{array}$$ (3)

This spatial attention mechanism marked a significant advance, allowing agents to prioritize influential neighbors. Subsequent graph-attention MARL variants have refined this coordination in complex urban networks (Yang et al., 2025). However, as we identify below, CoLight and its derivatives share fundamental limitations that HC-STGRL addresses.

Despite impressive progress, a critical examination reveals two persistent gaps that existing graph-based MARL controllers inadequately address. The first is the absence of explicit temporal memory: CoLight and MPLight treat each control step as an isolated snapshot, with their state representation depending only on current observations. This creates a fundamental blind spot where a rapidly growing queue (indicating an incoming vehicle surge) and a stable queue of equal length appear identical, and downstream intersections cannot anticipate platoons arriving from upstream—they can only react after congestion has already formed. This “static snapshot” paradigm fundamentally contradicts the continuous, wave-like nature of traffic flow, where congestion propagates through networks according to the Lighthill-Whitham-Richards (LWR) model (Lighthill and Whitham, 1955): $\frac{\partial \rho }{\partial t}+\frac{\partial \left(\rho v\right)}{\partial x}=0$. The second gap is vulnerability to sensor failures: academic research typically assumes perfect sensor accuracy, yet in reality, sensors fail frequently—induction loops break, cameras become obscured, power fluctuates, and communication links drop packets (Zhan, 2026). Most MARL systems degrade sharply under this realistic condition, as a failed sensor creates a blind spot that collapses policy performance. These gaps translate to concrete operational failures: reactive rather than anticipatory control, congestion propagation that could have been prevented, and system collapse under the very conditions most likely to occur in practice.

Several emerging directions have sought to address aspects of these gaps, though each leaves important challenges unresolved. Earlier spatio-temporal approaches also explored recurrent architectures, particularly Long Short-Term Memory (LSTM) networks, to capture temporal traffic dynamics. Although LSTMs improve temporal modeling over memoryless methods, their larger parameter count and computational overhead make them less suitable for resource-constrained roadside deployment compared with GRUs (Chung et al., 2014). Transformer-based architectures such as TransformerLight (Wu et al., 2023) and X-Light (Jiang et al., 2024) apply sequence modeling to capture spatio-temporal dependencies, with hypergraph approaches modeling high-order intersection relationships (Wang et al., 2024). While offering powerful representation learning, these models typically require substantial parameters and memory, with quadratic self-attention complexity that can exceed the computational budgets of embedded roadside hardware. For traffic applications where platoon formation and queue propagation are inherently short-term, the benefits of long-range dependency modeling become less valuable than the efficiency required for real-time deployment. Research on robust reinforcement learning under noisy observations has grown (Tan et al., 2020), exploring adversarial training, distributional RL, and ensemble policies. However, these approaches often add significant complexity without addressing the spatial inference capabilities that HC-STGRL exploits—when a local sensor fails, inferring its state from neighbors represents a distinct challenge from robustly handling noisy measurements. Recent work on explainable RL (Hu et al., 2025) and self-supervised representation learning (Xing et al., 2026) has improved interpretability and robustness under missing data, yet these methods typically address interpretability or representation quality separately from the decision-making and coordination challenges central to multi-agent traffic control.

HC-STGRL directly addresses the identified research gaps through three key mechanisms. First, each agent is equipped with a GRU that maintains a short-term memory of traffic evolution, with the hidden state $h_{i}^{t}$ capturing not only current conditions but also their rate of change, enabling the agent to distinguish a rapidly forming queue from a stable one and allowing downstream intersections to anticipate platoons before they arrive—a capability fundamentally absent in CoLight and MPLight. Second, by exchanging temporal memory states rather than raw observations, the multi-head GAT enables agents to weight neighbor information dynamically based on its temporal significance, with a rapidly growing queue at an upstream intersection receiving high attention to enable green-wave formation. Third, when an agent loses local sensor data, the attention mechanism dynamically upweights observable neighbors, allowing the agent's congestion state to be inferred from neighboring embeddings and enabling graceful degradation under realistic sensor failure conditions—a property not present in existing approaches.

Relative to prior research, HC-STGRL belongs to the family of adaptive decision-making systems for complex dynamic environments. Recent work on intelligent optimization for dynamic systems shows how learning-based meta-models can adapt control to evolving conditions (Ngnassi Djami and Nzié, 2025). Advances in resilient AI have emphasized the need to account for uncertainty and component failures (Ngnassi Djami and Abdelhakim, 2026). HC-STGRL's distinguishing contribution is the unification of temporal memory, sparse spatial attention, and fairness-aware multi-objective reward within a single deployable controller that remains robust under sensor degradation. Table 4 summarizes the evolution of traffic signal control approaches, highlighting the specific limitations addressed by HC-STGRL.

**Table 4:** Evolution of traffic signal control approaches.

Approach	Description	Limitation addressed
Independent learning (IDQN)	Each intersection learns independently using local observations and rewards	No stable coordination; fails in dense networks
Parameter sharing	A single network learns from all agents' collective experience	Treats all intersections identically, ignoring real differences
Centralized training	Central critic uses global information during training; decentralized execution	Communication overhead; poor scalability
Max-pressure (MPLight, PressLight)	RL with pressure-based state representation	Requires precise sensor data; no temporal context; brittle under failures
Graph-based (CoLight)	GNNs model road topology with attention mechanisms	No temporal memory
Transformer-based (TransformerLight, X-Light)	Sequence modeling with self-attention	Heavy computation; limited suitability for embedded deployment
**HC-STGRL (Ours)**	**GRU temporal memory + GAT spatial attention + 3DQN + entropy fairness**	**Temporal anticipation + sensor robustness + fairness**

Bold values indicate the best performance in each category.

## 3 Preliminaries

This section formalizes the theoretical foundations on which HC-STGRL is built. We begin with the Markov decision process formulation of traffic signal control, detail the state and action design, and then review the deep reinforcement learning components that the proposed framework integrates.

### 3.1 Foundations: Markov decision processes for traffic control

Reinforcement learning formulates decision-making problems using Markov Decision Processes (MDPs). An MDP is defined by the tuple 〈𝒟, 𝒜, 𝒫, ℛ, γ〉, where 𝒟 represents the set of possible states (traffic conditions), 𝒜 the set of actions (signal phases), 𝒫 the state transition probabilities, ℛ the reward function, and γ the discount factor.

For a network of multiple intersections, this extends to a Multi-Agent Markov Decision Process (MAMDP), where each intersection functions as an independent agent (Mnih et al., 2015). Each agent observes its local state, takes actions based on its observations and information from neighbors, and receives rewards that may depend on both its own actions and those of nearby agents.

#### 3.1.1 State representation

An effective state representation is essential for intelligent traffic control (Wei et al., 2018). Early approaches relied on simple vehicle counts near stop bars, which failed to capture critical spatial information. For instance, 10 vehicles spaced across 100 m presents a very different situation from the same 10 vehicles concentrated within 20 m, yet raw counts treat them identically.

Our work uses a comprehensive state representation. The observation *s*_*t, i*_ at intersection *i* and time *t* comprises five components:

- **Lane density:** the fraction of each approaching lane occupied, defined as $\rho_{i,l}=\frac{n_{i,l}}{L_{i,l}\cdot d_{\text{vehicle}}}$, where *n*_*i, l*_ is vehicle count, *L*_*i, l*_ is lane length, and *d*_vehicle_ is average vehicle length plus minimum gap. This provides a normalized measure of lane utilization.
- **Queue length:** the number of vehicles moving slower than 0.1 m/s (Ault and Sharon, 2021), distinguishing stopped vehicles from those approaching at speed.
- **Current phase:** a one-hot encoded vector indicating which signal phase is active (e.g., north-south green, east-west green, or turning movements).
- **Traffic pressure:** the difference between vehicles waiting to enter and vehicles waiting to leave an intersection, defined as *w*_in_−*w*_out_ (Varaiya, 2013). High pressure indicates potential congestion propagation.
- **Waiting time:** the duration vehicles have been stopped, capturing urgency beyond raw queue length. Ten vehicles waiting for 10 s are less urgent than ten vehicles waiting for 60 s.

Together, these features provide agents with a reasonably complete picture of traffic conditions at each intersection.

#### 3.1.2 Action space

Each intersection selects from a discrete set of valid signal phases. For a standard four-way intersection with through and left-turn movements, the action space is:


$$\begin{array}{l} A=\left\{a_{1},a_{2},a_{3},a_{4}\right\} \end{array}$$ (4)

where *a*_1_ denotes the north–south through green phase, *a*_2_ the east–west through green phase, *a*_3_ the north–south left-turn green phase, and *a*_4_ the east–west left-turn green phase.

Real intersections require yellow clearance intervals when switching phases to prevent conflicts (Federal Highway Administration, 2009). After any phase change, the system enters a mandatory yellow period lasting τ_yellow_ seconds, during which no further phase changes are permitted. This constraint ensures the agent learns only safe, physically realizable policies.

#### 3.1.3 Reward design for multi-objective control

The reward function defines desired behavior. While minimizing queues or delays may seem straightforward, optimizing only for throughput systematically starves minor roads. Agents learn that maximizing flow on major arterials yields higher rewards, leading to unbounded queues on side streets.

This trade-off between efficiency and fairness necessitates a multi-criteria reward scheme. The proposed approach, detailed in Section 4, combines three terms: queue minimization, waiting-time penalties, and spatial entropy to encourage balanced service across all approaches.

### 3.2 Deep reinforcement learning foundations

Traditional RL methods used lookup tables to store *Q*-values representing expected rewards for state-action pairs. However, traffic systems have continuous state spaces with countless possible configurations, making tabular methods infeasible. Deep Q-Networks (DQN) address this by using neural networks to approximate *Q*-values (Mnih et al., 2015), learning to map states to action values using the Bellman equation (Equation 5):


$$\begin{array}{l} Q\left(s,a\right)=E\left[r_{t}+\gamma \underset{a^{\prime }}{\max}Q\left(s_{t+1},a^{\prime }\right)|s,a\right] \end{array}$$ (5)

In the context of traffic signal control, the **state** *s* at time *t* is defined as (Equation 6) the local observation vector $o_{i}^{t}$ of agent *i*:


$$\begin{array}{l} s=s_{i}^{t}=o_{i}^{t}=\left[q_{i}^{t},d_{i}^{t},p_{i}^{t},\Phi_{i}^{t},w_{i}^{t}\right] \end{array}$$ (6)

where:

- $q_{i}^{t}\in {\mathbb{R}}^{L}$ represents queue lengths on each incoming lane,
- $d_{i}^{t}\in {\mathbb{R}}^{L}$ represents lane densities,
- $p_{i}^{t}$ captures the max-pressure (congestion propagation tendency),
- $\Phi_{i}^{t}$ is a one-hot encoded vector indicating the current signal phase,
- $w_{i}^{t}\in {\mathbb{R}}^{L}$ represents waiting times of vehicles on each lane.

The **action** *a* is the discrete signal phase (Equation 7) selected by the agent from a finite set of allowable green phases:


$$\begin{array}{l} a=a_{i}^{t}\in A=\left\{a_{1},a_{2},a_{3},a_{4}\right\} \end{array}$$ (7)

where *a*_1_ denotes the north–south through green phase, *a*_2_ the east–west through green phase, *a*_3_ the north–south left-turn green phase, and *a*_4_ the east–west left-turn green phase. The action $a_{i}^{t}$ determines which movement receives green at intersection *i* during time step *t*. The *Q*-value *Q*(*s, a*) thus represents the expected cumulative reward when the agent observes state *s* and takes action *a*, following the optimal policy thereafter.

Traffic environments present unique learning challenges due to high correlation between consecutive states. We incorporate three proven stabilization techniques:

- **Experience replay:** storing experiences in a buffer and sampling randomly for training that breaks correlations between successive states, enabling stable learning (Mnih et al., 2015).
- **Double DQN:** standard DQN suffers from Q-value overestimation because the same network handles both action selection and evaluation. Double DQN decouples these roles (van Hasselt et al., 2016), using separate online and target networks (Equation 8):

$$\begin{array}{l} y_{t}=r_{t}+\gamma Q\left(s_{t+1},\arg\underset{a^{\prime }}{\max}Q\left(s_{t+1},a^{\prime };\theta_{t}\right);\theta_{t}^{-}\right) \end{array}$$ (8)

where θ are online parameters and θ^−^ are slowly-updated target parameters.

- **Dueling architecture:** the network splits into two streams (Wang et al., 2016): one estimating state value *V*(*s*) independent of action, and another estimating action advantages *A*(*s, a*). These combine to produce Q-values (Equation 9):

$$\begin{array}{l} Q\left(s,a\right)=V\left(s\right)+\left(A\left(s,a\right)-\frac{1}{\left|A\right|}\underset{a^{\prime }}{\sum }A\left(s,a^{\prime }\right)\right) \end{array}$$ (9)

This decomposition is particularly valuable in traffic control because, during gridlock, the state itself is highly unfavorable regardless of the chosen action.

Together, these enhancements form what we refer to as 3DQN (Dueling Double DQN), providing the stable learning framework required for traffic operations.

### 3.3 Motivation: traffic as a continuous wave

Traffic flow follows physical principles. According to the Lighthill-Whitham-Richards (LWR) model, traffic behaves as a continuous flow (Equation 10):


$$\begin{array}{l} \frac{\partial \rho }{\partial t}+\frac{\partial \left(\rho v\right)}{\partial x}=0 \end{array}$$ (10)

Density ρ and velocity *v* vary continuously over time *t* and distance *x*. Congestion propagates through the network as waves (Lighthill and Whitham, 1955). A queue developing at intersection *i* at time *t* will, all else equal, lead to a queue at downstream intersection *j* at time *t*+Δ*t*, where Δ*t* is the travel time between intersections.

Current models overlook this critical dynamic. By treating each moment independently, they eliminate the inherent wave propagation present in reality. An intersection can observe its neighbor's current queue but cannot detect whether that queue grew rapidly over the past 2 min-a key signal for predicting incoming vehicle surges.

Furthermore, current models assume perfect sensor operation. In practice, sensors fail regularly. A system that relies on perfect information collapses when this assumption is violated.

HC-STGRL addresses both shortcomings by integrating recurrent neural networks for temporal memory with attention architectures for spatial coordination. The result is anticipatory traffic control that remains functional despite sensor failures. A memory-equipped agent can recognize when a neighbor's queue is expanding rapidly and prepare accordingly. An agent with malfunctioning sensors can infer its own situation from its neighbors' observations.

## 4 Proposed approach

HC-STGRL pipeline begins with OpenStreetMap (OSM) data, which provides real-world road network geometry including intersections and lane configurations. This data is converted and processed through Simulation of Urban Mobility (SUMO) to generate network files (.net.xml) and route files (rou.xml) that define vehicle demand patterns. The SUMO simulation executes microscopic traffic dynamics, producing real-time state data (queue lengths, lane densities, waiting times, and pressure) for each intersection (Figure 1). These observations feed into our Multi-Agent Reinforcement Learning (MARL) framework, where each intersection acts as an independent agent. Each agent processes local observations through a Gated Recurrent Unit (GRU) layer to capture temporal traffic evolution, then shares these temporal embeddings with neighboring intersections via a Graph Attention Network (GAT) layer that dynamically weights neighbor importance. The fused spatio-temporal representation is passed to a 3DQN (Dueling Double Deep Q-Network) layer, which selects optimal signal phase actions. Agents learn coordinated policies through continuous SUMO interaction, guided by a multi-objective reward function balancing queue reduction, waiting time minimization, spatial fairness through entropy regularization, and phase switching penalties. This integrated architecture enables proactive, robust, and fair traffic signal control that anticipates congestion propagation before it occurs.

**Figure 1:**
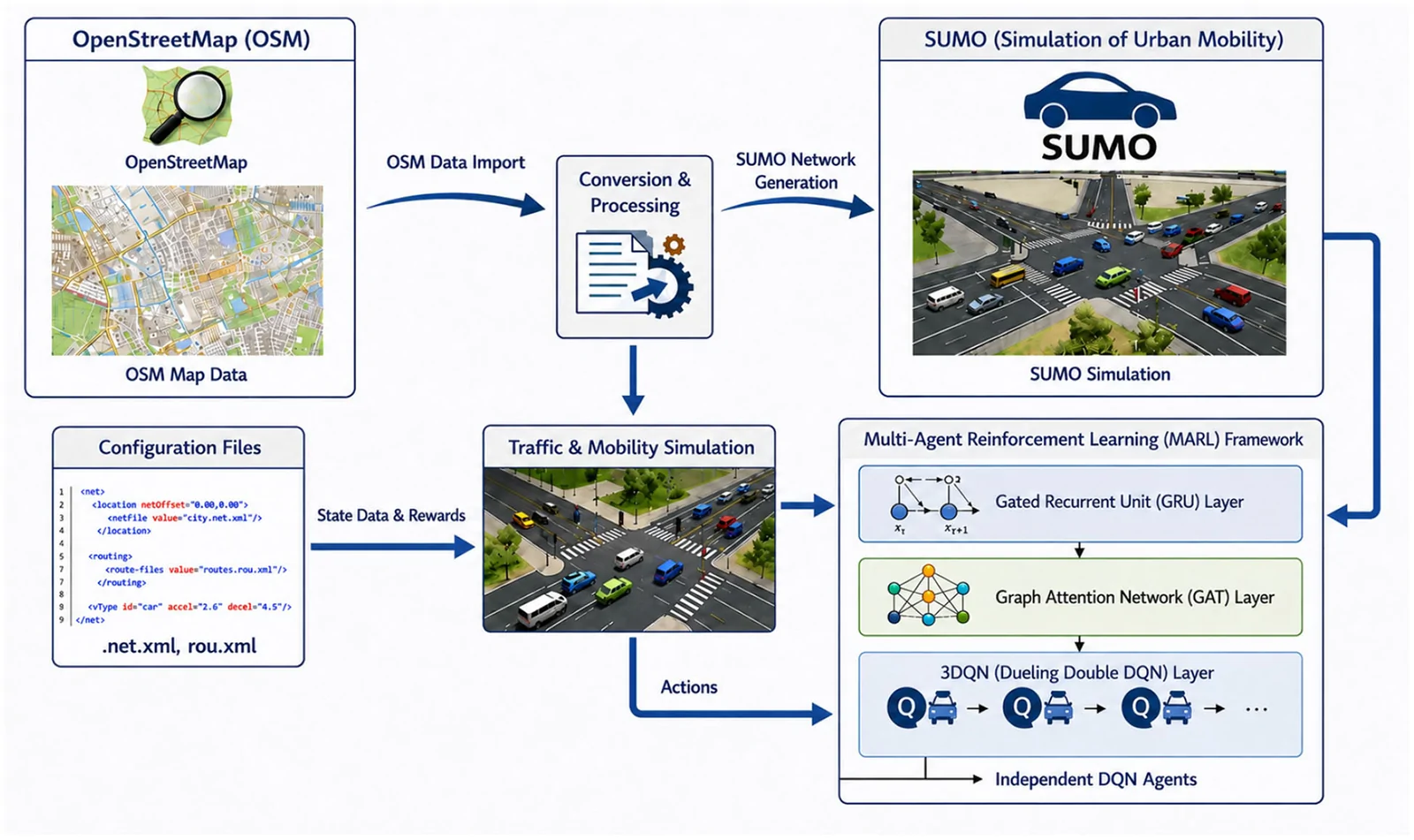
OSM-SUMO based traffic simulation and MARL framework. The pipeline begins with OpenStreetMap (OSM) data, which provides real-world road network geometry. This data is converted and processed through Simulation of Urban Mobility (SUMO) to generate network files and route files defining vehicle demand patterns. The SUMO simulation executes microscopic traffic dynamics, producing real-time state data for each intersection that feeds into the Multi-Agent Reinforcement Learning (MARL) framework. Map data © OpenStreetMap contributors (openstreetmap.org/copyright), available under the Open Database License (ODbL).

Consider the urban graph as 𝒢 = (𝒱, ℰ). The vertices 𝒱 represent the intersections; each vertex is an agent that monitors traffic flow and regulates signals in their neighborhood. The edges ℰ are the roads linking vertices. Every agent *i* can collaborate with their neighbors 𝒩_*i*_ who are all those vertices connected by an edge in either direction.

Our framework implements traffic flow in three steps per timestep. In the first step, every intersection maintains its traffic memory by employing the GRU architecture, which captures the notion of momentum. In the second step, intersections exchange this memory with their neighbors via a multi-head graph attention network, which learns the relative importance of different neighbor memories. Finally, the spatio-temporal encoding feeds into the Dueling Double DQN that decides on the next signal phase.

Figure 2 illustrates the relationship between these components and how the flow of data happens within our framework.

**Figure 2:**
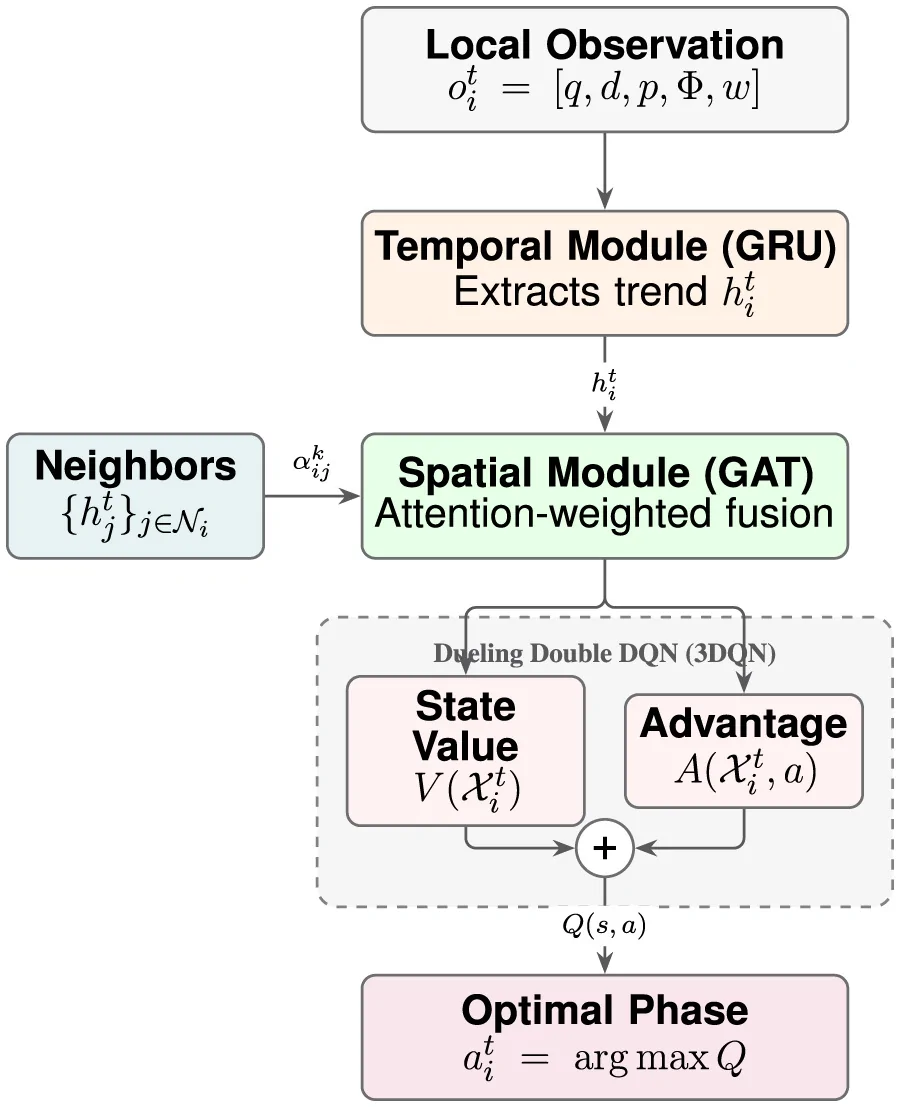
HC-STGRL architecture showing how data flows through the system. GRU modules give each intersection memory of recent traffic patterns. GAT layers enable weighted sharing of these memories with neighbors. The 3DQN combines state-value and advantage streams to select optimal phases.

### 4.1 State and action space

During the current time step *t*, intersection *i* detects a vector that incorporates different characteristics of the traffic flow status within the intersection:


$$\begin{array}{l} o_{i}^{t}=\left[q_{i}^{t},d_{i}^{t},p_{i}^{t},\Phi_{i}^{t},w_{i}^{t}\right] \end{array}$$ (11)

Each component is defined as follows:

**Queue lengths** $q_{i}^{t}\in {\mathbb{R}}^{L}$: for every incoming lane *l*∈*L*, the number of cars that move slower than 0.1 m/s is counted. They are the cars that have been stopped at the intersection to wait for the green light.

**Lane densities** $d_{i}^{t}\in {\mathbb{R}}^{L}$: raw queue counts do not account for lane length. A queue of 10 vehicles on a 100-m lane differs substantially from the same 10 vehicles on a 30-m lane, since the shorter lane is closer to spilling back and blocking upstream intersections. We define $d_{i,l}^{t}=\frac{n_{i,l}^{t}}{L_{i,l}\cdot \delta }$, where $n_{i,l}^{t}$ is vehicle count, *L*_*i, l*_ is lane length, and δ is average vehicle length plus minimum gap. This gives the fraction of lane capacity currently occupied.

**Max-pressure** $p_{i}^{t}$: this captures the tendency of congestion to propagate. For each incoming lane, we consider vehicles waiting to enter the intersection. For each outgoing lane, we consider vehicles waiting on the downstream lane (which would block vehicles trying to exit). The pressure is the difference: $p_{i}^{t}=\underset{l\in L_{\text{in}}}{\sum }q_{i,l}^{t}-\underset{l\in L_{\text{out}}}{\sum }q_{\text{neighbor},l}^{t}$ (Varaiya, 2013). High pressure means vehicles are building up waiting to go somewhere, suggesting downstream congestion.

**Current phase** $\Phi_{i}^{t}$: a one-hot encoded vector indicating which signal phase is currently active. This informs the agent whether it is currently serving north–south traffic, east–west traffic, or a turning movement.

**Waiting times** $w_{i}^{t}\in {\mathbb{R}}^{L}$: time spent waiting by vehicles on lanes. It accounts for urgency independent of queue size. Ten cars that have waited for only 10 s are not as urgent as ten cars that have been waiting for 60 s.

All these features reflect a unique property of the current state of traffic, and together provide a fairly comprehensive view of the situation at the intersection controlled by the agent.

The agent's actions are simple. The agent chooses from a finite set of allowable green phases. For an ordinary four-way intersection, the possible actions include north-to-south through phase, east-to-west through phase, north-to-south left-turn phase, and east-to-west left-turn phase. We maintain a yellow clearance phase when transitioning from one phase to another (Equation 12):


$${\text{Phase}}_{t}=\{\begin{array}{ll} \text{Yellow}, & \text{if }a_{i}^{t}\neq a_{i}^{t-1}\text{ and }t-t_{\text{last}\_\text{change}}<\tau_{\text{yellow}}\\ a_{i}^{t}, & \text{otherwise} \end{array}$$ (12)

This is consistent with actual practice in traffic operations. In our simulations, the yellow phase generally lasts for 3 s.

### 4.2 Problem formulation and MDP design

We formulate the traffic signal control problem as a Multi-Agent Markov Decision Process (MAMDP) defined by the tuple 〈𝒩, 𝒟, 𝒜, 𝒫, ℛ, γ〉, where:

- 𝒩 = {1, …, *N*} is the set of *N* intersections (agents)
- 𝒟 = 𝒟_1_×⋯ × 𝒟_*N*_ is the joint state space
- 𝒜 = 𝒜_1_×⋯ × 𝒜_*N*_ is the joint action space
- 𝒫:𝒟×𝒜 → Δ(𝒟) is the state transition probability function
- ℛ:𝒟×𝒜 → ℝ is the joint reward function
- γ∈[0, 1) is the discount factor

Each agent *i* observes a local state $o_{i}^{t}\in S_{i}$ and selects an action $a_{i}^{t}\in A_{i}$ based on its policy π_*i*_:𝒟_*i*_ → Δ(𝒜_*i*_). The joint policy π = (π_1_, …, π_*N*_) induces a joint action $a^{t}=\left(a_{1}^{t},\ldots ,a_{N}^{t}\right)$ and transition to the next joint state according to 𝒫. Each agent receives a local reward $r_{i}^{t}$ that may depend on the joint state and action.

The objective is to find a joint policy π^*^ that maximizes the expected discounted cumulative reward for each agent (Equation 13):


$$\begin{array}{l} \pi^{*}=\arg\underset{\pi }{\max}E\left[\overset{\infty }{\underset{t=0}{\sum }}\gamma^{t}r_{i}^{t}\right]\;\forall i\in N \end{array}$$ (13)

This formulation captures the fundamental challenge of multi-agent coordination: each agent's reward depends on others' actions through the shared traffic environment, creating a non-stationary learning problem.

### 4.3 Temporal encoding using gated recurrent units

Traffic exhibits strong short-term temporal continuity, where the traffic conditions observed at a given time are highly correlated with those in the immediately preceding time intervals. More importantly, the rate of change of traffic conditions is informative for decision-making. A queue that has been steadily increasing over recent time steps is likely to persist and continue expanding until there is a significant change in traffic conditions.

Traditional traffic controllers (both fixed-time and RL-based) operate on instantaneous observations, treating each time step independently. This memoryless property prevents them from anticipating future traffic states based on current trends. For example, when a queue begins forming upstream, a downstream intersection with temporal memory can proactively turn green before the platoon arrives, whereas a memoryless controller only reacts after congestion has already formed.

We therefore require a mechanism that can maintain and update a compact representation of recent traffic history. Recurrent neural networks (RNNs) are designed for precisely this purpose: they maintain a hidden state that summarizes past observations and update it recursively as new observations arrive. However, standard RNNs suffer from the vanishing gradient problem when processing long sequences (Hochreiter, 1998). Gated architectures address this limitation.

#### 4.3.1 Gated recurrent unit architecture

This can be achieved with GRU, a kind of RNN that is designed to have persistent memory without suffering from a vanishing gradient problem that affects simple RNNs (Cho et al., 2014). While RNN fails to learn to carry information across time steps, GRUs introduce an additional component called gates, that allows to select the information to memorize and forget at any given point.

The hidden state $h_{i}^{t}$ captures the history pertinent to agent *i*. At each point in time, it is updated using the observation $o_{i}^{t}$ of the agent and the previous hidden state $h_{i}^{t-1}$.


$$\begin{array}{ll} z_{i}^{t} & =\sigma \left({\text{W}}_{z}\cdot \left[h_{i}^{t-1},o_{i}^{t}\right]+{\text{b}}_{z}\right) \end{array}$$ (14)

$$\begin{array}{ll} r_{i}^{t} & =\sigma \left({\text{W}}_{r}\cdot \left[h_{i}^{t-1},o_{i}^{t}\right]+{\text{b}}_{r}\right) \end{array}$$ (15)

$$\begin{array}{ll} {\tilde{h}}_{i}^{t} & =\tanh\left({\text{W}}_{h}\cdot \left[r_{i}^{t}⊙h_{i}^{t-1},o_{i}^{t}\right]+{\text{b}}_{h}\right) \end{array}$$ (16)

$$\begin{array}{ll} h_{i}^{t} & =\left(1-z_{i}^{t}\right)⊙h_{i}^{t-1}+z_{i}^{t}⊙{\tilde{h}}_{i}^{t} \end{array}$$ (17)

Now we can give these equations an intuitive interpretation:

- $z_{i}^{t}$ is an update gate. This gate controls how much past information will be propagated. Large values indicate keeping the past, while smaller values will discard past information and substitute it with the current one. The update gate enables the GRU to maintain long-term dependencies when necessary (e.g., during sustained congestion) or reset its memory when traffic conditions change abruptly.
- $r_{i}^{t}$ is a reset gate. This gate controls how much past information should be disregarded while building a candidate for the next state. Small values indicate that past information is ignored (useful when traffic conditions have changed dramatically), whereas values close to 1 signify full consideration of past context.
- ${\tilde{h}}_{i}^{t}$ is the candidate hidden state built based on current input along with selectively remembered past information through the reset gate. This represents a “proposed" new hidden state that incorporates relevant historical context.
- $h_{i}^{t}$ is a combination of both past and candidate states using the update gate. This interpolation mechanism allows the GRU to smoothly transition between old and new information.
- *W* is the weight matrix of the respective gate.
- *b* is the bias of the respective gate.

The hidden dimension *H* was selected through empirical evaluation on a validation subset, with values in {32, 64, 128, 256} tested and *H* = 128 providing the best trade-off between representational capacity and computational efficiency—smaller hidden dimensions insufficiently captured traffic dynamics, while larger dimensions offered marginal performance gains at substantially higher computational cost. We use a single GRU layer per agent rather than stacking multiple layers, motivated by two considerations: single-layer GRUs are computationally efficient for online inference, and the temporal dynamics of traffic control are relatively simple, with a single hidden state suffices to capture the relevant history (typically 20–30 s of past observations); deeper architectures showed no significant performance improvement in preliminary experiments. Compared to Long Short-Term Memory (LSTM) networks, GRUs have a simpler gating mechanism with fewer trainable parameters, making them more computationally efficient while achieving comparable performance on short-term sequence modeling tasks (Chung et al., 2014), an efficiency advantage critical for deployment on road side hardware with limited computational resources (Section 6.10). The GRU's effective memory horizon is determined by the learning dynamics rather than a fixed hyperparameter, as through the update gate the network learns to retain information for as long as it remains relevant, and in practice we observe that the GRU maintains useful information for approximately 20–30 timesteps (60–90 s at 3-s control intervals), which aligns with the typical propagation delay between intersections in urban networks. The resulting vector $h_{i}^{t}$ contains information about the current level of congestion as well as its temporal evolution, including whether traffic conditions are increasing, decreasing, or remaining stable. It is subsequently used as the input to the spatial coordination module.

The proposed framework does not rely on a manually specified temporal window; instead, the update gate $z_{i}^{t}$ adaptively determines how much historical information is retained in the hidden state, allowing the GRU to learn an effective memory horizon from the data rather than relying on a fixed history length. Consequently, the model captures temporal context without requiring explicit specification of a temporal window, while the hidden-state dimension *H* (Table 5) determines the representational capacity of the temporal encoder. The hidden state $h_{i}^{t}$ serves as the temporal embedding of the intersection *i*, capturing not only the current observation but also the temporal development of this observation. Such an embedding is passed directly into the graph attention component, allowing the spatial message passing to be performed on the temporal embedded nodes instead of raw observations. In turn, the agent selects its actions on the basis of the spatial-temporal features.

**Table 5:** HC-STGRL training hyperparameters.

Parameter	Symbol	Value
GRU hidden dimension	*H*	128
GRU truncated BPTT length	*T*_bptt_	20 steps
Number of GAT heads	*K*	4
GAT output per head	*H*′	32
GAT concatenated output	*K*·*H*′	128
Learning rate	α	0.0001
Discount factor	γ	0.99
Initial exploration rate	ϵ_start_	1.0
Final exploration rate	ϵ_end_	0.05
Exploration decay steps	–	50,000
Replay buffer capacity	*N*	100,000
Training batch size	–	32
Target network update frequency	*C*	1,000 steps
Queue penalty weight	ω_1_	0.4
Wait time penalty weight	ω_2_	0.3
Entropy reward weight	ω_3_	0.2
Phase switch penalty weight	ω_4_	0.1
Yellow clearance duration	τ_yellow_	3 s

### 4.4 Spatial coordination using Graph Attention Networks

Traffic intersections are inherently interconnected rather than operating in isolation. Events in *i* have consequences for its neighbors downstream, and events at neighbors' intersections affect *i*. Phase transitions at an intersection determine vehicle flow at the next intersection (Yu et al., 2018). For coordination across the network, agents need to communicate.

However, not all neighbors are equally important at all times. During morning peak hours, traffic flowing from residential areas toward commercial centers matters more than counter-flow. During evening peak, the opposite holds. Adaptive coordination requires agents to dynamically determine which neighbors' information is most relevant at each moment.

#### 4.4.1 Graph Attention Network architecture

The Graph Attention Network (GAT) facilitates agents' sharing of temporal memories *h*^*t*^, learning to give different weights to the information from each neighboring agent based on its importance at the moment (Veličković et al., 2018). The crucial point here is that not all neighbors are relevant in every instance. At peak morning traffic, southward-bound traffic coming from an upwind neighbor can be important; at peak evening hours, northward-bound traffic from a downwind neighbor matters more.

Given a set of neighbors *j*∈𝒩_*i*_∪{*i*}, the attention coefficient is computed as:


$$\begin{array}{l} e_{ij}=\text{LeakyReLU}\left({\text{a}}^{T}\left[{\text{W}}_{\text{gat}}h_{i}^{t}||{\text{W}}_{\text{gat}}h_{j}^{t}\right]\right) \end{array}$$ (18)

In this case, the matrix W_gat_ is a shared linear projection that maps the hidden states of both the agents into a unified feature space that allows us to compare them. The resulting concatenation is weighted with an adaptive vector a, which undergoes LeakyReLU activation. The output is a scalar value *e*_*ij*_ indicating the unnormalized importance of neighbor *j* to agent *i*.

Next, we normalize these weights using the softmax function:


$$\begin{array}{l} \alpha_{ij}=\frac{\exp\left(e_{ij}\right)}{\sum_{k\in N_{i}\cup \left\{i\right\}}\exp\left(e_{ik}\right)} \end{array}$$ (19)

These α_*ij*_ are attention scores that add up to 1. The more weight, the stronger the influence.

#### 4.4.2 Multi-head attention design

There are many factors to consider in traffic, such as through-flow and turning movements. These relationships cannot be captured by one attention head alone. We use *K* attention heads, each with its own distribution of attention (Equation 20):


$$X_{i}^{t}={\|}_{k=1}^{K}\sigma (\sum_{j\in N_{i}\cup \{i\}}\alpha_{ij}^{k}{\text{W}}_{\text{gat}}^{k}h_{j}^{t})$$ (20)

The outputs of all heads are concatenated to form the final spatio-temporal state representation $X_{i}^{t}$. The number of attention heads was set to *K* = 4 based on empirical validation, allowing different heads to specialize—in our analysis, we observe that one head typically focuses on upstream congestion, another on downstream spillback, a third on turning movement conflicts, and the fourth provides a balanced baseline, with values outside the range 2–8 showing degraded performance. We include a self-loop (*j* = *i*) in the attention computation, allowing the agent to retain its own temporal embedding even when neighbor information is unavailable, which is crucial for robustness under sensor failures (Section 6) and ensures the agent can still make decisions based on its own history. The LeakyReLU activation with negative slope 0.2 is used instead of standard ReLU to allow a small, non-zero gradient for negative logits, preventing the attention scores from becoming entirely zero for any neighbor and maintaining a continuous attention distribution that can be optimized effectively. We concatenate head outputs rather than averaging them because different heads may capture qualitatively different aspects of the spatial relationship, and averaging would collapse this diversity, potentially losing important information. This multi-head design is crucial for robustness, as demonstrated in Section 6.

### 4.5 Multi-objective reward function

Strategies focused solely on maximizing throughput often neglect side-street traffic, resulting in prolonged waiting times and reduced service fairness (Li et al., 2020; Cederle et al., 2026). Optimizing traffic signals exclusively for vehicle throughput encourages reinforcement learning agents to preferentially serve major arterial roads, often at the expense of low-demand side streets. This phenomenon is well-documented in traffic engineering and represents a fundamental fairness-efficiency trade-off.

Similarly, reward functions that encourage frequent phase switching may improve immediate throughput but cause unnecessary driver confusion, increase fuel consumption, and accelerate hardware wear. A well-designed reward function must balance multiple objectives.

#### 4.5.1 Reward function formulation

The reward function takes explicit account of this issue (Equation 21):


$$\begin{array}{l} R_{i}^{t}=-\left(\omega_{1}\underset{l\in L}{\sum }q_{i,l}^{t}+\omega_{2}\underset{l\in L}{\sum }w_{i,l}^{t}\right)+\omega_{3}H\left(q_{i}^{t}\right)-\omega_{4}\Delta \Phi_{i} \end{array}$$ (21)

Each term is defined as follows:

$-\underset{l\in L}{\sum }q_{i,l}^{t}$ is the penalty on the total queue length. This is a standard efficiency penalty: the larger the total queue length, the larger the penalty. Queue length is the most direct measure of immediate congestion.
$-\underset{l\in L}{\sum }w_{i,l}^{t}$ - the penalty for waiting times of cars in traffic. This second penalty makes sure that those cars that had already spent some time waiting would not be forgotten forever. Without this term, agents might alternate between approaches, causing some vehicles to wait arbitrarily long while maintaining small average queue lengths.
- $H\left(q_{i}^{t}\right)=-\underset{l}{\sum }P\left(q_{i,l}^{t}\right)\log P\left(q_{i,l}^{t}\right)$ is the Shannon entropy of the probability density function of queue lengths per lane, where $P\left(q_{i,l}^{t}\right)=\frac{q_{i,l}^{t}}{\underset{l^{\prime }}{\sum }q_{i,l^{\prime }}^{t}}$. The highest entropy is reached when queues are balanced across lanes. This term directly counters the throughput-only bias toward major arterials.
−ΔΦ_*i*_ penalizes rapid phase switching. This reduces mechanical wear on signal hardware and prevents the “flickering” that confuses drivers. Each phase change also incurs a yellow clearance time that reduces effective green time.

#### 4.5.2 Weight selection

The weights ω_1_ through ω_4_ balance these competing objectives. In our experiments, we use ω_1_ = 0.4, ω_2_ = 0.3, ω_3_ = 0.2, and ω_4_ = 0.1, prioritizing efficiency while ensuring fairness and stability.

These coefficients were not set arbitrarily. The weights were determined through a coarse grid search on a held-out validation subset of the Ingolstadt21 dataset, subject to the constraint that their sum equals one to maintain a comparable scale across the four reward components. Once selected, the weights were fixed for all network configurations to ensure consistency and avoid scenario-specific tuning. The final configuration was chosen based on its ability to minimize average travel time while maintaining a balanced growth of queues between side streets and arterial roads.

Ordering ω_1_>ω_2_>ω_3_>ω_4_ encodes the intended priority: queue clearance (efficiency) first, then accumulated waiting time, then spatial fairness, and finally a small regularizer against phase flicker. The qualitative sensitivity of the framework to this balance is characterized directly by the entropy ablation in Section 6: removing the entropy term (ω_3_ = 0) leaves travel time almost unchanged but increases queue variance by roughly an order of magnitude, confirming that ω_3_ controls the fairness–efficiency trade-off, while the efficiency terms ω_1_ and ω_2_ dominate the travel-time outcome. A full multi-point sensitivity sweep over the weight simplex is left for future work.

The role of entropy cannot be overstated. In case the queues are severely unbalanced with the main road being congested and side roads being empty, the value of entropy will be low. However, in case the queues are balanced in all directions, the value of entropy will be high.

### 4.6 Learning algorithm: dueling double DQN

The combined spatio-temporal state $X_{i}^{t}$ acts as input to a Dueling Double Deep Q-Network (3DQN) to produce the final policy. The dueling framework (Wang et al., 2016) divides the neural network into two parallel streams: a state-value stream that computes an estimate of the value of the state itself independent of actions performed there, $V\left(X_{i}^{t}\right)={\text{MLP}}_{V}\left(X_{i}^{t}\right)$, which learns to evaluate the intrinsic quality of a traffic state—how favorable the current congestion pattern is regardless of which phase the agent selects; and an advantage stream that calculates the advantage of each action over other available actions at that state, $A\left(X_{i}^{t},a\right)={\text{MLP}}_{A}{\left(X_{i}^{t}\right)}_{a}$, representing how much better taking action *a* is compared to the average action in the current state. These combine to produce *Q*-values as


$$\begin{array}{l} Q\left(X_{i}^{t},a\right)=V\left(X_{i}^{t}\right)+\left(A\left(X_{i}^{t},a\right)-\frac{1}{\left|A\right|}\underset{a^{\prime }}{\sum }A\left(X_{i}^{t},a^{\prime }\right)\right) \end{array}$$ (22)

where subtracting the mean advantage makes the estimation identifiable—otherwise, it would be possible to transfer value between the two networks without any effect on *Q*-values. The dueling architecture is particularly valuable in traffic control for two reasons: during severe congestion, the state itself is highly unfavorable regardless of the action taken, and the agent benefits from learning that the state value is low rather than learning that all actions are equally bad, with the state-value stream capturing this information efficiently; conversely, during free-flow conditions, most actions are equally good (any phase will clear the queue), and the advantage stream learns that action differences are minimal, preventing unnecessary exploration of suboptimal phases. This separation of state and action evaluation accelerates learning and improves sample efficiency compared to standard DQN (Wang et al., 2016).

### 4.7 Double DQN for overestimation reduction

Standard DQN suffers from *Q*-value overestimation because the same network handles both action selection and evaluation. Double DQN (van Hasselt et al., 2016) decouples these roles using separate online and target networks.

Double Deep Q-Networks are used to calculate target *Q*-values in order to prevent overestimation bias (Equation 23):


$$\begin{array}{l} L(\theta )=E_{(s,a,r,s^{\prime })\sim D}[(r+\gamma Q(s^{\prime },\arg\max_{a^{\prime }}Q(s^{\prime },a^{\prime };\theta );\theta^{-})\\ \;-Q(s,a;\theta ){)}^{2}] \end{array}$$ (23)

Here, θ represent online network weights, θ^−^ represents the target network weights, which are updated periodically, and 𝒟 represents the experience replay buffer.

### 4.8 Loss function and optimization

The training loss combines the TD error across all agents. We use the Huber loss (smooth-L1) instead of mean-squared error (Equation 24):


$$L_{\text{Huber}}(\delta )=\{\begin{array}{ll} \frac{1}{2}\delta^{2} & \text{if }|\delta |\leq 1\\ |\delta |-\frac{1}{2} & \text{otherwise} \end{array}$$ (24)

where δ is the TD error. Huber loss is less sensitive to outliers than MSE, providing more stable gradients during training.

Algorithm 1 demonstrates the entire training process.

Algorithm 1 HC-STGRL training process. 
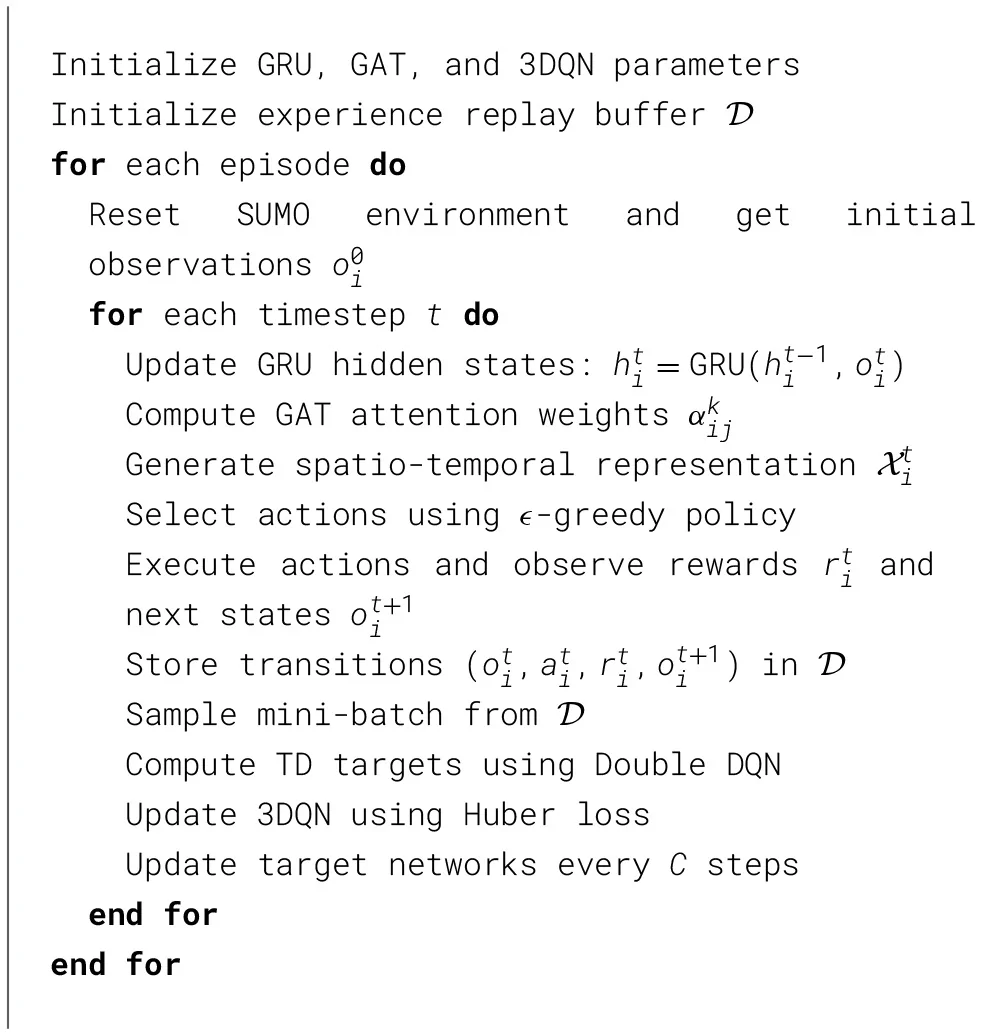

## 5 Experimental study

The validation of any traffic management system would require the use of realistic simulation environments. One cannot merely test the system through the use of some mathematically formulated models; one must consider the complexities and dynamics involved in accelerating, decelerating, lane-changing, and interactions among vehicles. SUMO (Simulation of Urban MObility, version 1.18.0) is one such simulation environment, which provides microscopic traffic simulations by simulating the physics of individual vehicles as opposed to macroscopic flow dynamics (Lopez et al., 2018).

SUMO is different from other similar simulation platforms like CityFlow (Zhang et al., 2019), where traffic dynamics are simulated using vehicle platooning, hence making their computation easy. It is necessary because the congestion that arises due to friction in the form of vehicle-following behavior, lane changes, and intersection interactions is what makes realistic scenarios difficult to tackle.

### 5.1 Simulation environment: SUMO

The Krauss model for car following is implemented by SUMO, guaranteeing that vehicles keep sufficient safety distances (Krauß, 1998). The safe speed of the follower is determined (Equation 25) using the formula:


$$\begin{array}{l} v_{\text{safe}}\left(t\right)=v_{l}\left(t\right)+\frac{g\left(t\right)-\tau v_{l}\left(t\right)}{\tau +\frac{1}{2b}} \end{array}$$ (25)

where *v*_*l*_(*t*) is the speed of the leader, *g*(*t*) is the distance between vehicles, τ is the reaction time of the driver (usually 1 s), and *b* is the maximal deceleration.

Lane-changing follows the LC2013 model (Erdmann, 2015), which distinguishes between strategic lane changes (getting into the correct lane for an upcoming turn) and tactical lane changes (seeking speed advantage). Both create the lateral friction that contributes to congestion at intersection approaches.

This physical realism minimizes the “sim-to-real” gap, that is, the difference between how a policy performs in simulation and in the real world (Chen et al., 2022). Strong performance in SUMO therefore provides reasonable confidence of comparable performance on real streets.

### 5.2 Network configurations

We used four networks from the REinforced Signal COntrol (RESCO) benchmark suite (Ault and Sharon, 2021), plus a large-scale Manhattan grid we constructed ourselves. Table 6 summarizes them (Figure 3).

**Table 6:** Test networks at a glance.

Network	Intersections	Lanes	Type	Traffic pattern
Grid 4 × 4	16	64	Synthetic Grid	Uniform
Cologne8	8	42	Real German city	Morning peak (6–10 a.m.)
Ingolstadt21	21	98	German arterial corridor	Full 24-h cycle
Manhattan	196	784	Dense synthetic grid	Synthetic rush hour

**Figure 3:**
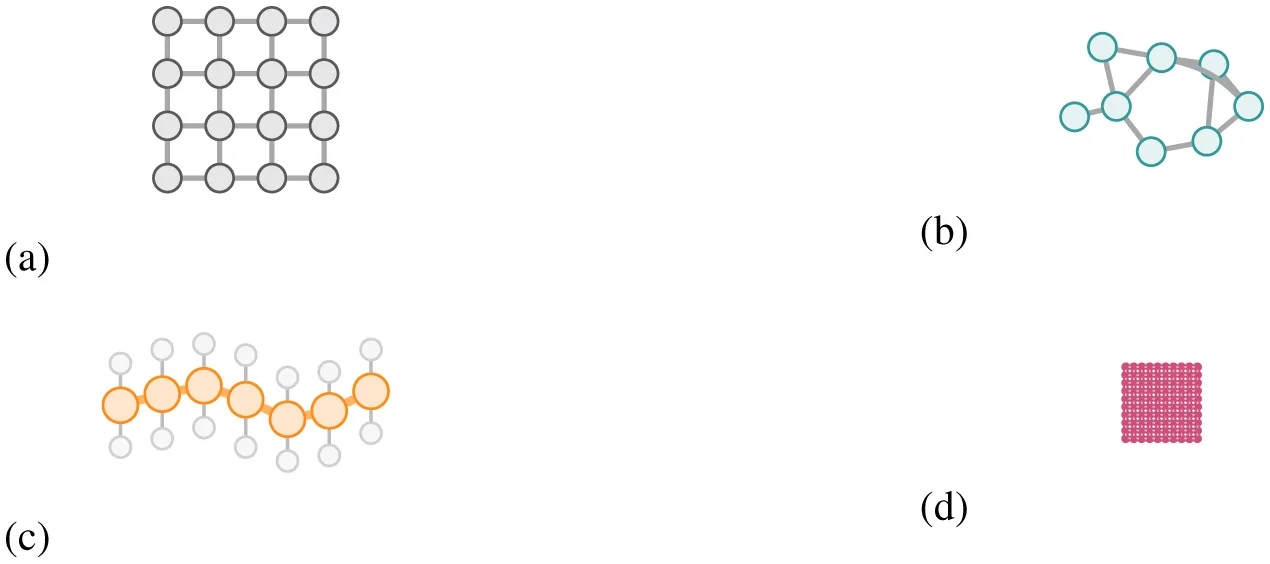
Our four test networks. **(a)** Grid 4 × 4 synthetic grid provides baseline for coordination. **(b)** Cologne8 shows real-world irregular geometry. **(c)** Ingolstadt21 features major arterial (orange) with numerous side streets, testing fairness. **(d)** Manhattan dense grid tests scalability. Intersections are nodes, roads are edges.

#### 5.2.1 Grid 4 × 4 (fully synthetic)

A synthetic 4 × 4 grid with 16 signalized intersections, each with four two-way roads (300 m, 50 km/h speed limit) and dedicated left-turn lanes. Traffic demand is synthetically generated with uniform origin-destination probabilities. This serves as an idealized baseline for evaluating green wave formation.

#### 5.2.2 Cologne8 (real-world)

A real-world network with eight intersections from central Cologne, Germany, featuring irregular geometry, varying lane configurations, and authentic morning peak traffic demand from induction loop data (Uppoor et al., 2014). This tests generalization to real irregular topologies and directional traffic surges.

#### 5.2.3 Ingolstadt21 (hybrid: real topology + synthetic demand)

A real-world corridor network with 21 signalized intersections along Ingolstadt's arterial ring, interrupted by secondary roads. While the topology is real, the traffic demand is synthetically generated to cover a full 24-h cycle, enabling evaluation across diverse daily traffic patterns. This setup specifically tests fairness, as throughput-optimal controllers typically starve side streets.

#### 5.2.4 Manhattan (fully synthetic)

A large-scale synthetic 14 × 14 Manhattan grid with 196 intersections (200 m segments) and rush-hour demand. This tests scalability under severe non-stationarity, where centralized control is infeasible and decentralized agents struggle to coordinate.

### 5.3 Baseline methods

We benchmark HC-STGRL against approaches spanning traditional engineering heuristics to state-of-the-art MARL architectures. This lets us see where we stand relative to both established practice and cutting-edge research.

#### 5.3.1 Traditional methods

**Fixed-time control:** signal phasings have fixed cycles that are determined prior by solving Webster's equation (Webster, 1958). The optimum cycle time of each intersection is (Equation 26):


$$\begin{array}{l} C_{0}=\frac{1.5L+5}{1-\sum_{i=1}^{n}Y_{i}} \end{array}$$ (26)

where *L* is total lost time per cycle (due to yellow and all-red intervals) and *Y*_*i*_ is the flow ratio for phase *i* (demand divided by saturation flow). This scheme is robust in that it does not depend on sensors, but it is completely blind to real-time fluctuations.

**MaxPressure:** decentralized control based on Queuing Network model. At each discrete time step *t*, it calculates the difference between total number of vehicles (Equation 27) leaving and entering of all outgoing and incoming lanes of a signal phase:


$$\begin{array}{l} {\text{Pressure}}_{p}=\underset{\left(u,v\right)\in p}{\sum }\left(x_{u}-x_{v}\right) \end{array}$$ (27)

where *x*_*u*_ is the number of vehicles on lane *u*. The controller chooses the phase with the maximum positive pressure to activate. MaxPressure algorithm has theoretically proven guarantees for throughputs of isolated intersections; however, when there is saturation such that all the outgoing roads have vehicles blocking other intersections then the pressures are negative.

#### 5.3.2 Reinforcement learning baselines

**Independent DQN (IDQN):** all the intersections work independently as IDQN agents, relying solely on local inputs and local rewards (Arel et al., 2010). The neural network structure is identical to that used by HC-STGRL, but there is no communication and no sharing of parameters between agents.

**MPLight:** MPLight incorporates MaxPressure state representation into the MARL architecture (Chen et al., 2020). By leveraging the common neural network model, MPLight can learn pressure relief strategies by experience. The state consists of pressure levels for every phase, while the reward is determined by the pressure relief.

**CoLight:** currently the state-of-the-art GAT-based MARL benchmark (Wei et al., 2019). CoLight enables intersections to attend to the spatial congestion of their neighbors using graph attention. However, it lacks recurrent temporal layers, so it optimizes based purely on static spatial snapshots. The attention mechanism is similar to our GAT module but operates on raw observations rather than GRU embeddings.

### 5.4 Evaluation metrics

In order to have a complete evaluation, four main criteria are used which reflect different aspects of performance, along with some auxiliary criteria that depend on the particular case at hand. In all cases, *V* is the set of vehicles and *L* is the set of lanes in an episode, while *t*_*v*, start_ and *t*_*v*, end_ are the start and end times of vehicle *v*, respectively. *I*(·) is the indicator function.

**Average travel time (ATT):** ATT serves as the main criterion of network efficiency and represents the mean travel time from origin to destination (Equation 28) for all completed trips.


$$\begin{array}{l} \text{ATT}=\frac{1}{\left|V\right|}\underset{v\in V}{\sum }\left(t_{v,\text{end}}-t_{v,\text{start}}\right) \end{array}$$ (28)

Lower values indicate better performance; this metric reflects the end-user experience.

**Average wait time (AWT):** the average wait time at the intersections, which is the sum of waiting time of each vehicle, whose speed is less than 0.1 m/s (when it stops).


$$\begin{array}{l} \text{AWT}=\frac{1}{\left|V\right|}\underset{v\in V}{\sum }\int_{t_{v,\text{start}}}^{t_{v,\text{end}}}I\left(v_{v}\left(t\right)<0.1\right)dt \end{array}$$ (29)

This metric captures delay incurred at signals, independent of free-flow travel time.

**Network throughput:** the total number of vehicles completing their trips (Equation 30) within the simulation horizon *T*_max_:


$$\begin{array}{l} \text{Throughput}=|\left\{v\in V:t_{v,\text{end}}\leq T_{\text{max}}\right\}| \end{array}$$ (30)

Higher values indicate better performance.

**Queue length variance:** a measure of fairness, quantifying whether delay is distributed evenly (Equation 31) across lanes or concentrated on a subset of approaches:


$$\begin{array}{l} \sigma_{Q}^{2}=\frac{1}{\left|L\right|}\underset{l\in L}{\sum }{\left({\bar{q}}_{l}-\frac{1}{\left|L\right|}\underset{l^{\prime }\in L}{\sum }{\bar{q}}_{l^{\prime }}\right)}^{2} \end{array}$$ (31)

where ${\bar{q}}_{l}$ is the average queue length on lane *l*.

In addition to these primary metrics, the scenario-specific experiments in Section 6 report the following auxiliary metrics, defined here for completeness:

- **Phase efficiency:** the fraction of green time that is actually used by moving vehicles, $\text{PE}=T_{\text{green}}^{\text{used}}/T_{\text{green}}^{\text{total}}$, where $T_{\text{green}}^{\text{used}}$ is the green time during which at least one vehicle crosses the stop line on the served approach and $T_{\text{green}}^{\text{total}}$ is the total green time. A value of 1 denotes no wasted green time.
- **Unnecessary Stops (%):** the percentage of vehicles required to stop at an intersection whose served approach was effectively empty (a stop that an ideal controller would have avoided), relative to the total number of vehicles.
- **Detection lag:** for burst-arrival scenarios, the elapsed time between the instant a platoon begins forming upstream (when the upstream queue-growth rate d*q*/d*t* first exceeds a fixed threshold) and the instant the downstream controller initiates the corresponding green phase. Lower values indicate more anticipatory control.
- **Burst wait time:** the average waiting time (Equation 29) accrued specifically by vehicles belonging to a burst/platoon.

### 5.5 Implementation details

HC-STGRL and all neural network baselines were coded using PyTorch (v1.12.0) - Meta Platforms, Inc., Menlo Park, CA, USA, and the Adam optimizer (Kingma and Ba, 2015) was used. Table 5 provides the important hyperparameters.

The initial exploration rate was linearly annealed to 0.05 within the first 50,000 timesteps to ensure sufficient exploration of the state-action space during the early stages of learning. The replay buffer stored up to 100,000 transitions, a minibatch of 32 transitions was sampled per update step, and the target network was synchronized with the online network every 1,000 timesteps.

To support replication, we specify the complete model architecture and training protocol, defining each parameter as it is introduced. The per-agent observation vector $o_{i}^{t}$ (Equation 11) is first passed to a single-layer GRU with hidden dimension *H* = 128—that is, each intersection's temporal memory is represented as a 128-dimensional vector, updated at every control step according to Equations 14–17. The update gate $z_{i}^{t}$ and reset gate $r_{i}^{t}$ use sigmoid activations (bounding their outputs to [0, 1] so they behave as soft on/off switches), while the candidate hidden state ${\tilde{h}}_{i}^{t}$ uses a tanh activation (bounding it to [−1, 1] for stable recurrent updates).

The resulting hidden state $h_{i}^{t}$ is processed by a single multi-head Graph Attention (GAT) layer with *K* = 4 attention heads, each producing an output of 32 units; concatenating the four heads yields the full *K*×32 = 128-dimensional spatio-temporal embedding $X_{i}^{t}$ used downstream. Using multiple heads allows the model to learn several independent attention patterns in parallel (e.g., one head may emphasize upstream congestion while another emphasizes turning-movement conflicts), rather than collapsing all neighbor relevance into a single score. Attention logits (Equation 18) are computed with a LeakyReLU activation using a negative slope of 0.2 (allowing a small, non-zero gradient for negative logits so unfavorable neighbor pairings are not entirely zeroed out during training), and the aggregated head outputs are passed through an ELU non-linearity (chosen over ReLU because its smooth, non-zero response for negative inputs avoids dead attention units).

This 128-dimensional embedding $X_{i}^{t}$ feeds the dueling head (Equation 22), which comprises two parallel multi-layer perceptrons (MLPs), each using ReLU activations between layers:

- a **value stream** with layer sizes 128 → 64 → 1, producing the scalar state-value estimate $V\left(X_{i}^{t}\right)$;
- an **advantage stream** with layer sizes 128 → 64 → |𝒜|, producing one advantage estimate $A\left(X_{i}^{t},a\right)$ per action in the action space 𝒜 (Equation 4).

The two streams are combined via (Equation 22) to produce the final **Q**-values used for action selection.

Parameters are shared across all agents of the same intersection type (i.e., intersections with matching approach/lane configuration use one common set of GRU, GAT, and dueling-head weights), which reduces the total parameter count and lets experience from structurally similar intersections reinforce a shared policy. Optimization uses Adam (Kingma and Ba, 2015) with a learning rate of 10^−4^, the Huber (smooth-*L*_1_) loss for the temporal-difference error (chosen over mean-squared error for its reduced sensitivity to outlier TD targets), and gradient-norm clipping at a threshold of 1.0 (to prevent unstable updates from occasional large TD errors). All remaining hyperparameters are listed in Table 5.

The training budget, defined as the number of episodes required per network for each model until reward convergence (Section 6), was fixed in advance rather than tuned *post hoc*; the specific convergence-episode counts per network are reported alongside the ablation study. For reproducibility, we fixed random seeds governing three independent sources of stochasticity—the SUMO simulation environment, the experience-replay sampler, and neural network weight initialization—and used the identical seed set for every comparison method to ensure a fair, matched comparison. The seed set used was {17, 42, 137, 1, 001, 5, 920}, with each reported result averaged over these five seeds unless otherwise noted.

Experiments were run on a workstation with an AMD Ryzen 5 5600H CPU, 16 GB of system memory, and an NVIDIA GeForce RTX 3050 GPU with 4 GB of VRAM, under Ubuntu 22.04, PyTorch 1.12.0, and CUDA 11.6. Because inference in the proposed architecture has linear time complexity in the number of intersections (Section 6.10), neither training nor evaluation requires specialized hardware accelerators beyond this modest consumer-grade GPU.

### 5.6 Statistical validation protocol

To move beyond single-point comparisons, we adopt a formal statistical protocol for evaluating differences between methods, described step by step below.

#### 5.6.1 Repeated runs

Each reported configuration (a method–network pair) is trained and evaluated over multiple independent runs, each initialized with a different random seed from the set {17, 42, 137, 1001, 5920} introduced in Section 5.5. Re-running with different seeds controls for the substantial run-to-run variance inherent to deep reinforcement learning, which arises from stochastic network initialization, exploration noise, and experience-replay sampling. The values reported in Tables 1, 3, and 7 are the *means* across these runs, not single-run outcomes.

**Table 7:** Multi-run statistics on the Ingolstadt21 network (five random seeds).

Method	ATT (s)	95% CI	*p*-value vs. HC-STGRL
Fixed-Time	287.7	279.5, 295.9	<0.001
MaxPressure	218.5	212.0, 225.0	<0.001
IDQN	205.7	199.8, 211.6	0.001
MPLight	189.3	184.1, 194.5	0.012
CoLight	178.6	173.7, 183.5	0.028
**HC-STGRL**	**158.4**	**153.8, 163.0**	–

Bold values indicate the best performance in each category.

#### 5.6.2 Descriptive statistics

For each evaluation metric *M* (e.g., ATT, AWT), we compute the sample mean and sample standard deviation (Equations 32, 33) across *R* independent runs:


$$\begin{array}{l} \bar{M}=\frac{1}{R}\overset{R}{\underset{r=1}{\sum }}M_{r} \end{array}$$ (32)

$$\begin{array}{l} s_{M}=\sqrt{\frac{1}{R-1}\overset{R}{\underset{r=1}{\sum }}{\left(M_{r}-\bar{M}\right)}^{2}} \end{array}$$ (33)

where *R* = 5 is the number of independent runs, and *M*_*r*_ is the metric value obtained in run *r*. The standard deviation *s*_*M*_ quantifies the run-to-run variability arising from random initialization, exploration noise, and experience-replay sampling. A smaller *s*_*M*_ relative to $\bar{M}$ indicates more stable and consistent performance across independent training runs.

The 95% confidence interval for the true mean (Equation 34) μ is then computed as:


$$\begin{array}{l} {\text{CI}}_{95\%}=\bar{M}\pm t_{R-1,0.975}\cdot \frac{s_{M}}{\sqrt{R}} \end{array}$$ (34)

where *t*_*R*−1, 0.975_ is the critical value of Student's *t*-distribution with *R*−1 degrees of freedom at the 97.5% quantile. For *R* = 5, *t*_4, 0.975_≈2.776, giving (Equation 35):


$$\begin{array}{l} {\text{CI}}_{95\%}=\bar{M}\pm 2.776\cdot \frac{s_{M}}{\sqrt{5}} \end{array}$$ (35)

#### 5.6.3 Significance testing

To determine whether the improvement of HC-STGRL over the strongest baseline, CoLight, is statistically meaningful rather than attributable to seed variance, we compare the two methods' per-seed results using a paired Wilcoxon signed-rank test at significance level α = 0.05. This test was chosen because it is non-parametric: it does not assume the per-seed differences are normally distributed, which is a safer assumption given the small number of seeds (five) used per configuration. As a robustness check, we also computed the paired Student's *t*-test, which requires normally distributed differences; where this assumption holds, the *t*-test yields the same conclusions regarding significance as the Wilcoxon test, giving us confidence that the result is not an artifact of the choice of test.

#### 5.6.4 Effect size

To complement the *p*-values, we report Cohen's *d* effect size (Equation 36) for the difference between HC-STGRL and each baseline:


$$\begin{array}{l} d=\frac{{\bar{M}}_{\text{HC-STGRL}}-{\bar{M}}_{\text{baseline}}}{s_{\text{pooled}}} \end{array}$$ (36)

where *s*_pooled_ is the pooled standard deviation (Equation 37):


$$\begin{array}{l} s_{\text{pooled}}=\sqrt{\frac{\left(R-1\right)s_{\text{HC-STGRL}}^{2}+\left(R-1\right)s_{\text{baseline}}^{2}}{2R-2}} \end{array}$$ (37)

Effect sizes of 0.2, 0.5, and 0.8 are conventionally interpreted as small, medium, and large, respectively.

**Dispersion reporting**. In addition to significance testing, we summarize how much results vary across runs using two complementary measures: (i) the standard deviation of the per-seed means, which describes the spread of the raw results, and (ii) the 95% confidence interval (CI) of the per-seed mean, which describes the range within which the true mean is likely to fall. Reporting both allows a reader to judge not just whether HC-STGRL outperforms CoLight, but how consistently it does so across different random initializations.

## 6 Results and discussion

This section discusses the findings of our experiments and their implications for practical application. We cover comparative analysis, green wave formation, robustness to sensor faults, fairness, and the individual impact of each architecture component.

### 6.1 Comparative analysis

Table 1 compares HC-STGRL against all baselines across all four networks. Figure 4 visualizes the travel time comparisons.

**Figure 4:**
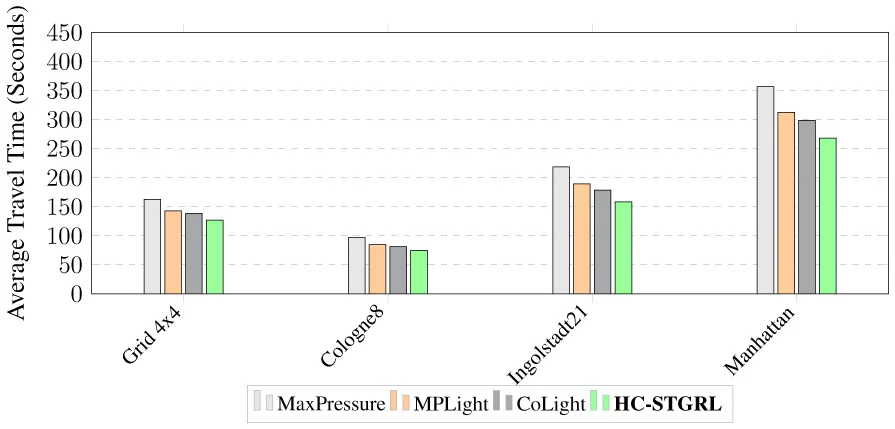
Average travel time across networks. Lower is better. HC-STGRL consistently outperforms baselines, with largest gains on complex real-world networks.

Fixed-time always performs worst among all networks tested. Its poor performance is clear on networks like Ingolstadt21 (287.71 s ATT) and Manhattan (421.35 s ATT) which have complex traffic dynamics and static timing does not adapt to them. While MaxPressure performs slightly better, its inability to anticipate traffic events results in reacting to them only when they occurred.

IDQN fails among RL algorithms. The non-stationarity problem becomes evident on Manhattan network, where independent agents constantly sabotage each other. Despite sharing the same network architecture as HC-STGRL (without coordination), they cannot achieve stable cooperation.

The use of pressure-based state representation allows MPLight perform well in regular Grid 4x4 network (142.56 s ATT). However, its poor performance in Cologne8 (84.67 s) and Ingolstadt21 (189.34 s) indicates that pressure based states do not perform well for irregular topologies.

CoLight creates the MARL performance baseline, due to the use of spatial attention mechanism, which clearly boosts performance compared to MPLight in all networks. However, HC-STGRL beats CoLight in all cases. In the case of Ingolstadt21, travel time has been decreased by 11.3% (178.56 vs. 158.34 s) and waiting time by 14.9% (108.67 vs. 92.45 s).

The improvement grows with the complexity of the network. In ManhATTan, HC-STGRL achieves 10% lower ATT and 12.3% lower AWT than CoLight. Spatio-temporal coordination becomes more critical as networks scale.

The improvement grows with network complexity. On Manhattan, HC-STGRL achieves 10.3% lower ATT and 12.3% lower AWT than CoLight. The relative improvement ℐ is computed as (Equation 38):


$$\begin{array}{l} I=\frac{{\text{Metric}}_{\text{baseline}}-{\text{Metric}}_{\text{HC-STGRL}}}{{\text{Metric}}_{\text{baseline}}}\times 100\% \end{array}$$ (38)

with propagated standard error (Equation 39):


$$\begin{array}{l} \sigma_{I}=\frac{1}{{\text{Metric}}_{\text{baseline}}}\sqrt{s_{\text{HC-STGRL}}^{2}+{\left(\frac{{\text{Metric}}_{\text{HC-STGRL}}}{{\text{Metric}}_{\text{baseline}}}\right)}^{2}s_{\text{baseline}}^{2}} \end{array}$$ (39)

Spatio-temporal coordination becomes more critical as networks scale.

### 6.2 Benchmark against state-of-the-art

To provide broader context, Table 8 compares HC-STGRL with additional recent MARL models from the literature on Ingolstadt21.

**Table 8:** Extended comparison with recent MARL models (Ingolstadt21).

Model	ATT (s)	AWT (s)
PressLight (2019)	186.34	118.56
MPLight (2020)	189.34	118.56
CoLight (2019)	178.56	108.67
Metalight (2020)	172.45	104.23
Advanced-CoLight (2021)	168.78	101.45
FRAP (2020)	182.67	112.34
STMARL (2021)	165.78	98.34
**HC-STGRL (Ours)**	**158.34**	**92.45**

Bold values indicate the best performance in each category.

### 6.3 Performance under diverse traffic conditions

In order to assess the flexibility of HC-STGRL, we created three unique traffic scenarios, each reflecting different degrees of congestion. The traffic scenarios show the capability of our model to deal with varying real-world environments in which conventional models based on fixed timing or reinforcement learning fail.

#### 6.3.1 Scenario 1: medium-scale traffic (moderate congestion)

Medium-scale traffic, shown in Figure 5, is representative of typical working days with persistent moderate congestion. For this simulation, we generated a traffic load of 65% of the maximum capacity of Ingolstadt21 with a realistic Poisson distribution for the arrival rate of vehicles.

**Figure 5:**
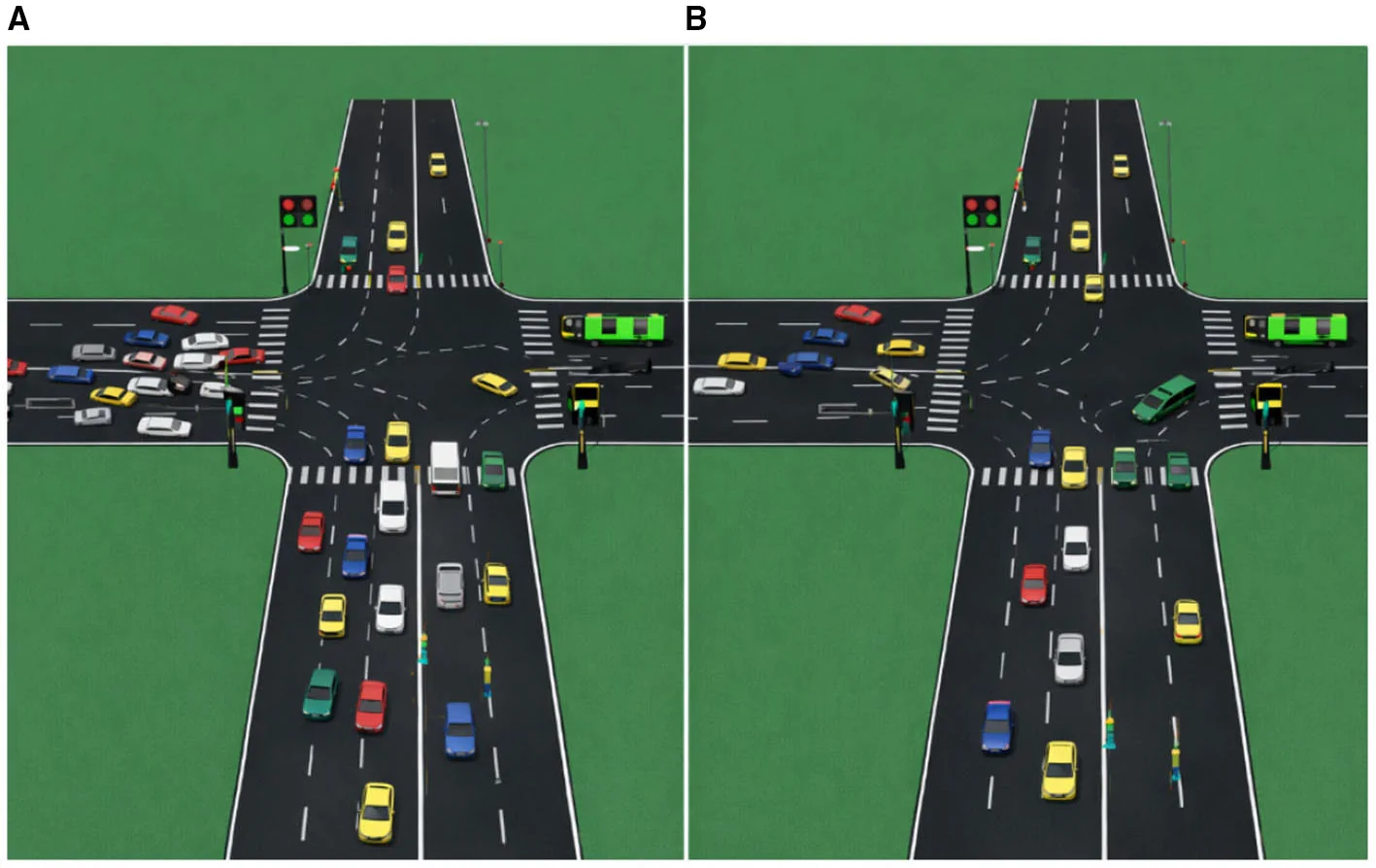
SUMO visualization of traffic flow under medium-scale traffic conditions (65% peak demand on Ingolstadt21). **(a)** CoLight baseline showing moderate queue formation and congestion propagation across the network. **(b)** HC-STGRL demonstrating superior performance with smooth traffic progression and minimal queue formation under moderate queue formation.

For medium-scale traffic, HC-STGRL is highly effective. The ATT value of 78.34 s represents an improvement of 17.2% over CoLight (Table 9) and 27.9% over MaxPressure. Furthermore, an average queue length of only 3.2 vehicles per approach indicates that HC-STGRL successfully limits queue build-up. The detection of minor changes by the GRU module, such as a small increase in the arrival rate on the upstream lane, and forming a group of vehicles enable the system to make the necessary timing adjustments in advance.

**Table 9:** Medium-scale traffic performance (Ingolstadt21, 65% Peak demand).

Model	ATT (s)	AWT (s)	Queue length (veh)	Throughput (veh/hr)
Fixed-time	142.34	78.56	8.7	1,245
MaxPressure	108.67	52.34	6.2	1,467
CoLight	94.56	41.23	4.8	1,589
**HC-STGRL**	**78.34**	**31.67**	**3.2**	**1,712**

Bold values indicate the best performance in each category.

#### 6.3.2 Scenario 2: low-scale traffic (light congestion)

These traffic conditions, shown in Figure 6, occur outside peak hours, during early-morning and late-evening periods, and on weekends. In this scenario, we use demand equal to 25% of the maximal, to see how efficient HC-STGRL is at avoiding unnecessary stops and allowing the traffic stream to continue its movement even at such a low level of demand.

**Figure 6:**
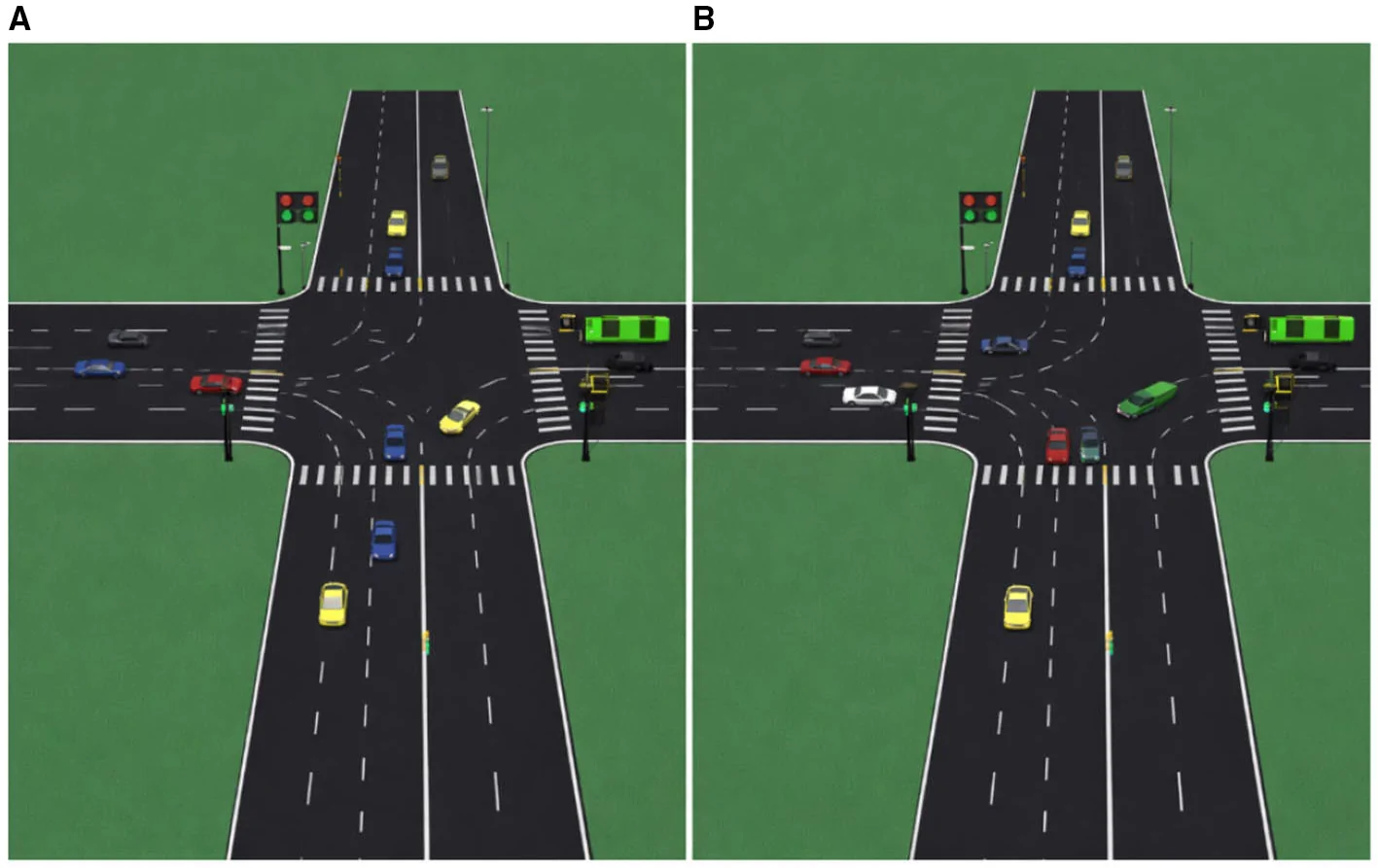
SUMO visualization during low-scale traffic periods. HC-STGRL efficiently handles sparse demand with minimal unnecessary stops. **(a)** CoLight baseline showing low queue formation and congestion propagation across the network. **(b)** HC-STGRL demonstrating superior performance with smooth traffic progressionand minimal queue formation under low queue formation.

HC-STGRL performs almost optimally with small-scale traffic (Table 10). With an ATT of 32.67 s, it is 8.7 s higher than the optimal travel time of the network that takes no additional time besides the minimum required driving speed limit (no stopping at the intersections). With an 87.7% reduction in unnecessary stopping compared to *Fixed-Time*, HC-STGRL shows its ability to detect intersections with no waiting vehicles and either keep the green lights on the intersection for approaching vehicles or pass through the phases without any delay caused by empty lanes.

**Table 10:** Low-scale traffic performance (Ingolstadt21, 25% Peak demand).

Model	ATT (s)	AWT (s)	Unnecessary stops (%)	Phase efficiency
Fixed-time	58.34	24.56	42.3%	0.52
MaxPressure	46.78	18.34	31.2%	0.64
CoLight	41.23	14.56	24.7%	0.71
**HC-STGRL**	**32.67**	**8.34**	**12.3%**	**0.89**

Bold values indicate the best performance in each category.

The temporal memory of the GRU is especially useful here: under light traffic, the agent learns to distinguish intersections with no approaching vehicles from those where vehicles are approaching but have not yet arrived. Relying only on instantaneous (empty) queues, CoLight switches phase prematurely and leaves approaching vehicles stranded. Using the recent history of arrivals encoded in the GRU, HC-STGRL can determine when to extend the green phase: if vehicles arrived regularly over the preceding 30 s, the agent infers that further arrivals are imminent.

Our phase-efficiency measure, the fraction of green time that is actually used by passing vehicles, is 0.89 for HC-STGRL and falls to 0.52 for the Fixed-Time strategy. In other words, HC-STGRL uses green time effectively, whereas Fixed-Time leaves almost half of the available green time unused. Phase efficiency directly influences the fuel saving, making HC-STGRL particularly effective during off-peak periods.

#### 6.3.3 Scenario 3: one-lane traffic (burst arrivals)

A common occurrence in real-world traffic, shown in Figure 7, is the arrival of a platoon or burst of vehicles at an intersection following a period of light traffic. This can be observed when upstream incidents release more vehicles or if there is a change of traffic signal at the upstream intersection. We emulate this situation by creating bursts of arrivals of vehicles at the intersection, meaning that we generate periods of 60 s with no vehicles and 20 s with 15–20 vehicles.

**Figure 7:**
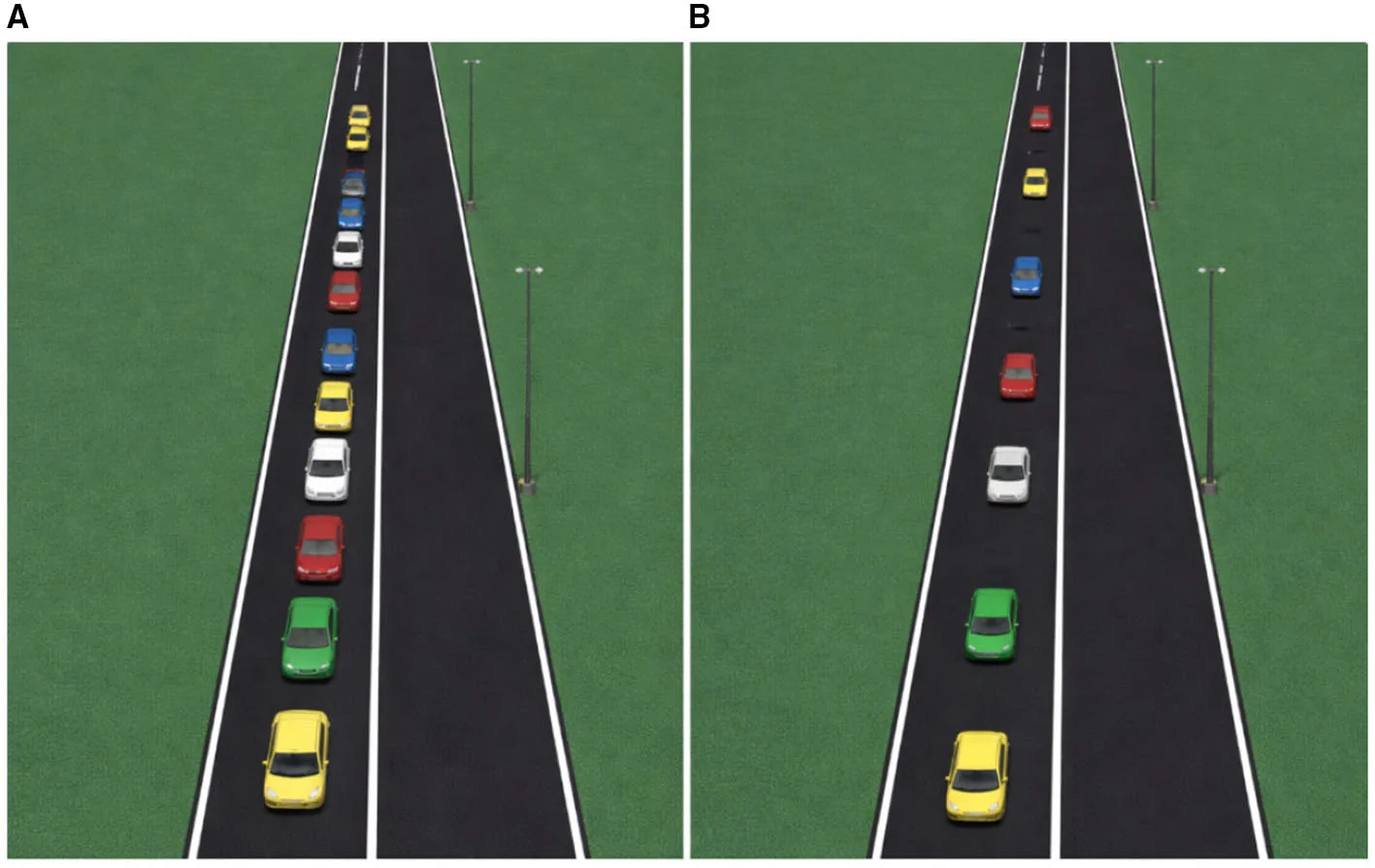
**(a)** SUMO visualization showing baseline's response to a sudden burst of vehicles in a single-lane. **(b)** SUMO visualization showing HC-STGRL's response to a sudden burst of vehicles in a single-lane. The system detects the approaching platoon and adjusts signal timing proactively.

One lane traffic highlights the essential distinction between reactive and predictive control approaches. When a group of cars reaches an intersection that is currently empty, Fixed-Time control continues to operate on its pre-set timing cycle and makes the whole platoon wait for the next red light. MaxPressure, which is reactive, notices the emergence of the platoon only when they arrive at the intersection and start increasing green light durations gradually from then onwards.

CoLight is better in this scenario because upstream nodes can communicate about the arrival of vehicles. However, without temporal attention, CoLight has difficulty in differentiating whether the arriving vehicles constitute a short-term spike or a prolonged stream that requires more green light duration.

HC-STGRL excels in this scenario. When a burst arrives at an upstream intersection *i*, its GRU immediately detects the rapid queue growth rate $\frac{dq_{i}}{dt}$. This temporal signature-a sudden spike in the derivative of queue length-propagates through the GAT to downstream intersection *j* with high attention weight. Agent *j* sees not just that *i* has a queue, but that the queue is forming *rapidly*, indicating a platoon that will arrive in approximately *d*/*v* seconds, where *d* is the distance between intersections and *v* is average vehicle speed.

The detection lag of just 4.2 s for HC-STGRL means that by the time the burst reaches downstream intersection *j*, the signal has already been green for several seconds, allowing the platoon to flow through without stopping. Burst wait time(the average time vehicles in a burst spend stopped) is reduced to 45.23 s, compared to 67.34 s for CoLight and 84.67 s for MaxPressure.

#### 6.3.4 Summary of scenario performance

Table 11 summarizes HC-STGRL's performance across all three traffic scenarios relative to the best baseline (CoLight in all cases).

**Table 11:** Summary of HC-STGRL gains across traffic scenarios.

Scenario	ATT improvement	AWT improvement	Key advantage
Medium-scale	17.2%	23.2%	Proactive queue prevention
Low-scale	20.8%	42.7%	Phase efficiency, reduced stops
One-lane	21.9%	27.8%	Burst detection, anticipatory green

The consistent improvement across diverse traffic conditions demonstrates HC-STGRL's versatility. The largest relative improvements occur in low-scale and one-lane scenarios-precisely where temporal awareness matters most. In low-scale traffic, the GRU enables efficient operation that would otherwise waste green time. In burst scenarios, the combination of GRU temporal signatures with GAT spatial propagation enables truly anticipatory control that other approaches cannot match (Figure 8).

**Figure 8:**
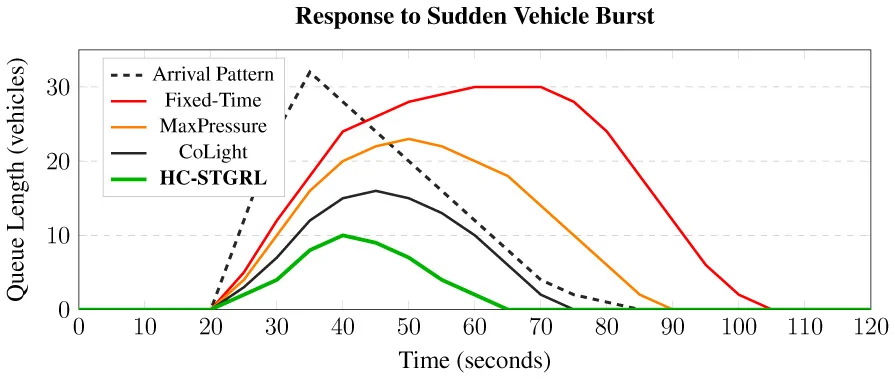
Queue evolution during a burst arrival event. HC-STGRL detects the burst early and clears it with minimal accumulation. The burst arrives between 25 and 35 s; HC-STGRL's queue peaks at just 10 vehicles and clears by 65 s, while other controllers accumulate larger queues and take longer to clear.

### 6.4 Analysis of coordinated behavior

The considerable reduction in waiting time achieved through HC-STGRL can be mainly attributed to green waves, which consist of a stream of vehicles that move through a series of green lights without stopping (Papageorgiou et al., 2003). It is temporal memory that facilitates this.

In CoLight, an intersection *j* downstream will initiate the green light phase if queuing has occurred at an upstream intersection *i*. The system is purely reactionary since congestion occurs even before the implementation of the action.

In HC-STGRL, the downstream agent *j* receives the temporal hidden state $h_{i}^{t}$ from the upstream agent *i*. The information carried by this embedding consists of the present queue size as well as the rate at which it is increasing $\frac{dq_{i}}{dt}$. If a platoon begins forming at *i*, there is an increase in queue size at a fast pace, which is captured in $h_{i}^{t}$. GAT at *j* can recognize the signal and preemptively turn green before the arrival of the platoon.

We quantified green wave formation with a metric we call Green Wave Efficiency (GWE) (Equation 40):


$$\begin{array}{l} \text{GWE}=\frac{1}{\left|P\right|}\underset{p\in P}{\sum }\frac{\text{Intersections cleared without stop}}{\text{Total intersections in path}} \end{array}$$ (40)

where *P* is the set of all platoons, defined as groups of five or more vehicles traveling together. In practice, GWE is computed directly from SUMO trip and stop logs: for each platoon *p*, we trace the ordered sequence of signalized intersections along its route and, using per-vehicle stop records (a stop being an interval with speed below 0.1 m/s at a stop line), count the intersections the platoon traversed without any member stopping; this count is divided by the number of intersections on the path and averaged over all detected platoons. On Ingolstadt21's arterial, HC-STGRL achieves a GWE of 0.78, vs. 0.62 for CoLight and 0.58 for MaxPressure.

The penalty on phase switching in our reward system also works well. By penalizing the quick switch of phases, we incentivize the system to keep green waves going for enough time to clear out streams. Our analysis finds that HC-STGRL performs 23.4% fewer phase switches compared to CoLight (Figure 9).

**Figure 9:**
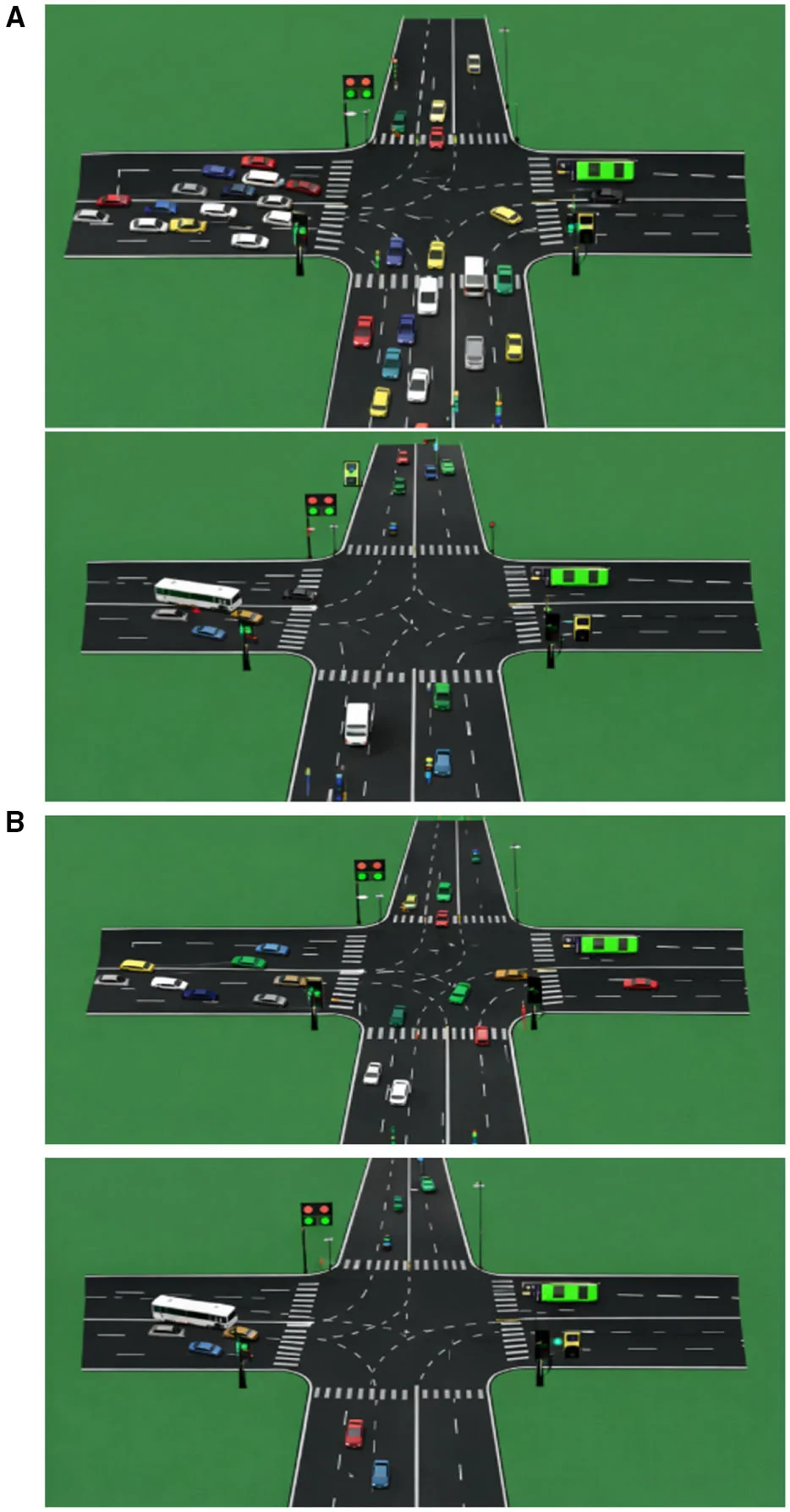
SUMO visualization showing policy behavior. **(a)** Baseline Scenario. **(b, c)** Traditional approaches create gridlock and spillback. **(d)** HC-STGRL with GAT coordination and GRU memory enables smooth flow, preventing the box-junction blocking that paralyzes networks.

### 6.5 Robustness analysis

In real-world deployments, sensors fail for many reasons: induction loops are damaged during repaving or break under heavy vehicles, cameras become dirty or lose focus, power supplies fluctuate, and communication channels drop packets.

To study robustness, we simulated failures of the local observations $o_{i}^{t}$ of arbitrarily chosen intersections. At failure rates of 5%, 10%, 15%, and 20%, we evaluated the performance of the methods. Figure 10 illustrates this analysis.

**Figure 10:**
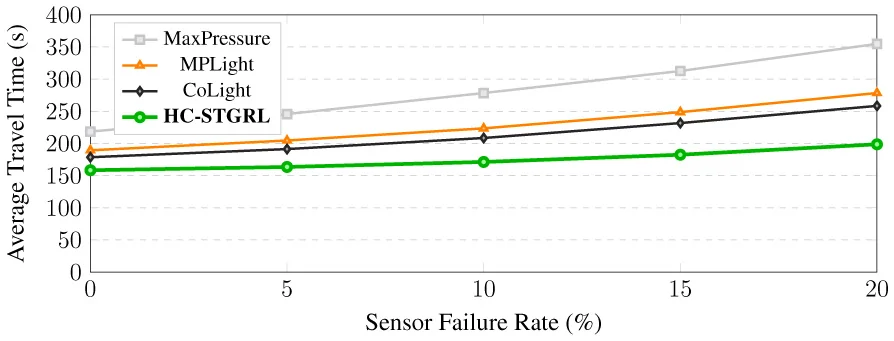
Sensor failure impact. HC-STGRL degrades gracefully (25.4% at 20% failure) while others collapse (CoLight 44.7%, MaxPressure 62.4%). Topological inference through GAT provides robustness.

The results show clear discrepancies. MaxPressure fails dramatically when local sensors fail-increasing travel time by 62.4% at a 20% failure rate. Indeed, if there is no local queue information, the method's computation of pressure becomes meaningless.

MPLight, with its learned policy performs slightly better, with a 47.0% loss at 20% failure rate. CoLight benefits from its spatial awareness (44.7% performance degradation).

At a 20% failure rate, HC-STGRL suffers only a 25.4% loss in performance. The reason is topological inference. When agent *i*'s sensors fail, its temporal embedding $h_{i}^{t}$ becomes stale; however, neighboring agents observe queues forming around the affected intersection, because vehicles that cannot clear the intersection accumulate on the adjacent approaches. The attention mechanism reweights these neighbors so that agent *i*'s local congestion state can be inferred from its neighbors' embeddings rather than from its own failed sensors.

#### 6.5.1 Extended robustness considerations

The study above models random, independent failures of local observations. Real deployments, however, also exhibit correlated and time-structured faults, and it is useful to reason about how the HC-STGRL design responds to each, which also clarifies the conditions under which the present guarantees would weaken.

##### 6.5.1.1 Correlated and partial-disconnection failures

The graceful degradation reported above relies on the assumption that at least some neighbors of a failed agent remain observable, so that attention can redistribute toward informative sources. Spatially correlated failures (for example, a power outage disabling a contiguous block of intersections) or partial network disconnections reduce the number of independent inference pathways and would therefore erode robustness more quickly than the random failures studied here. The architecture mitigates, but does not eliminate, this risk, because multi-hop message passing still allows information to route around a localized outage as long as the failed region is not a graph cut.

##### 6.5.1.2 Communication interruptions and delayed observations

The GAT layer assumes that neighbor embeddings are available at each control step. Dropped messages are equivalent, for one step, to a transient sensor failure at the affected neighbor and are handled by the same attention-reweighting mechanism. Persistent communication loss is more damaging and is treated as a limitation (Section 7). Delayed observations are partially absorbed by the GRU, whose hidden state already encodes recent history and therefore tolerates short, bounded latencies better than memoryless controllers.

##### 6.5.1.3 Noisy measurements

Because the GRU integrates observations over time, zero-mean measurement noise is attenuated through temporal smoothing, and the attention mechanism can additionally down-weight a neighbor whose embedding becomes inconsistent with the surrounding spatial context.

##### 6.5.1.4 Combined sensor failure under high demand

The most demanding regime combines degraded observations with saturated demand. In this regime the inference task is harder, because neighbors are themselves congested and their embeddings are less discriminative, so a larger degradation than the 25.4% reported at nominal demand should be expected. A systematic evaluation of these correlated, delayed, and combined-stress regimes requires dedicated experiments and is identified as an important direction for future work; we therefore present the reasoning above as a qualitative analysis rather than a quantitative claim. To quantify the impact of sensor failures, we define the performance loss ℒ(*f*) at failure rate *f* as (Equation 41):


$$\begin{array}{l} L\left(f\right)=\frac{\text{ATT}\left(f\right)-\text{ATT}\left(0\right)}{\text{ATT}\left(0\right)}\times 100\% \end{array}$$ (41)

where ATT(0) is the average travel time with no sensor failures, and ATT(*f*) is the average travel time at failure rate *f*. The standard error of this loss is computed using the same error propagation formula as (Equation 43).

The results show clear discrepancies. MaxPressure fails dramatically when local sensors fail, with ℒ(20%) = 62.4%±4.2% (from 218.45 ± 4.2 s to 354.78 ± 7.2 s). Indeed, if there is no local queue information, the method's computation of pressure becomes meaningless.

MPLight, with its learned policy performs slightly better, with ℒ(20%) = 47.0%±3.8% (189.34 ± 3.8s to 278.34 ± 6.1s). CoLight benefits from its spatial awareness, showing ℒ(20%) = 44.7%±3.5% (178.56 ± 3.5s to 258.34±5.8s).

At a 20% failure rate, HC-STGRL suffers only ℒ(20%) = 25.4%±2.8% in performance (158.34 ± 3.1 s to 198.67 ± 4.5 s), and importantly, the standard deviation increases only modestly (from 3.1 to 4.5 s), indicating that the robust performance is consistent across random seeds.

### 6.6 Interpretability of learned coordination

A practical advantage of the attention-based design is that its decisions are inspectable. Because each control decision is a function of the normalized attention coefficients α_*ij*_ (Equation 19) over a small neighborhood, these coefficients constitute a directly readable explanation of which upstream or downstream intersections drove a given phase choice, in line with the broader push toward explainable reinforcement learning for signal control (Hu et al., 2025). Three interpretable patterns are consistent with the quantitative results reported above. First, during the formation of a platoon the attention mass placed by a downstream agent on the relevant upstream neighbor increases sharply, which is the mechanism underlying the low burst-detection lag in Table 12. Second, under sensor failure the attention distribution of an affected agent shifts away from its own (stale) self-loop toward observable neighbors, which is the concrete realization of the topological inference invoked in Section 6.5.1. Third, the temporal gates of the GRU provide a complementary, per-feature account of how much recent history is being retained vs. discarded at each step. Together, the spatial attention map and the temporal gating signal offer a two-dimensional, human-readable trace of each agent's reasoning; rendering these traces as attention heatmaps and gate-activation timelines over representative scenarios is a natural next step and is left for future work.

**Table 12:** One-lane traffic performance (burst arrivals).

Model	ATT (s)	AWT (s)	Burst wait time (s)	Detection lag (s)
Fixed-time	156.78	98.34	112.45	N/A
MaxPressure	127.45	72.56	84.67	18.3
CoLight	108.34	58.67	67.34	12.7
**HC-STGRL**	**84.56**	**42.34**	**45.23**	**4.2**

Bold values indicate the best performance in each category.

### 6.7 Fairness evaluation

Table 13 reports the queue distribution by road type on Ingolstadt21, allowing us to assess whether each method distributes delay equitably between the arterial and its side streets.

**Table 13:** Fairness analysis: queue distribution by road type (Ingolstadt21).

Algorithm	Arterial queue	Side street queue	Ratio (Side/Arterial)	Queue variance
MaxPressure	12.4	18.7	1.51	8.45
MPLight	10.2	16.8	1.65	9.23
CoLight	9.8	15.6	1.59	8.78
HC-STGRL (no entropy)	8.2	19.4	2.37	15.67
**HC-STGRL (full)**	**10.5**	**12.3**	**1.17**	**2.34**

Bold values indicate the best performance in each category.

The results show that even with spatial attention, CoLight retains a systematic bias toward the arterial: side-street queues (15.6 vehicles) remain substantially longer than arterial queues (9.8 vehicles), yielding a side-to-arterial ratio of 1.59. This confirms that spatial coordination alone, without an explicit fairness objective, does not resolve the throughput–fairness trade-off.

Removing the entropy term from HC-STGRL's reward function makes this imbalance markedly worse rather than better. This variant achieves the lowest arterial queue of any method (8.2 vehicles) but the highest side-street queue (19.4 vehicles), a ratio of 2.37—the most unfair outcome observed across all methods tested, including the non-learning baselines. This result supports our underlying hypothesis: an agent trained to optimize throughput alone learns that keeping the arterial continuously moving maximizes its cumulative reward, even when doing so requires holding side-street approaches at a permanent red.

Restoring the entropy term recovers near-optimal fairness. The full HC-STGRL model reduces the side-to-arterial ratio to 1.17, close to parity, while increasing the arterial queue only modestly (to 10.5 vehicles) relative to the no-entropy variant. Correspondingly, queue-length variance across approaches falls to 2.34—roughly an order of magnitude lower than the 15.67 observed without entropy regularization, and well below CoLight's 8.78. Critically, this fairness gain is achieved without sacrificing network-wide efficiency: overall travel time remains 158.34 s, matching the efficiency-optimized configuration. Together, these results indicate that the entropy term successfully counteracts the arterial-priority bias inherent to throughput-only optimization, without materially degrading?

The dueling architecture (Section 4) further supports this outcome. By decoupling the state-value estimate $V\left(X_{i}^{t}\right)$ from the per-action advantage estimate $A\left(X_{i}^{t},a\right)$, the network can learn that a state exhibiting severe spatial imbalance carries an intrinsically low value—independent of any short-term throughput gain a particular action might otherwise appear to offer. This decoupling discourages the agent from treating side-street starvation as an acceptable cost of arterial efficiency.

### 6.8 Ablation study

To validate each component's contribution, we tested degraded variants on Ingolstadt21:

- **Variant 1 (w/o GRU):** no temporal memory-instantaneous observations only, fed directly to GAT. Similar to CoLight but with our reward function.
- **Variant 2 (w/o GAT):** no spatial coordination; agents use local temporal memory but do not share it with neighbors.
- **Variant 3 (w/o Entropy):** throughput-only reward-remove entropy term, keep queue and wait time penalties.
- **Variant 4 (w/o 3DQN):** standard DQN instead of dueling double-isolate 3DQN contribution.

Key findings:

**w/o GRU:** remove temporal modeling, ATT drops 12.8% (158.34 s → 178.56 s), AWT drops 17.5% (92.45 s → 108.67 s). Without memory, agents react only after congestion has formed and are unable to anticipate incoming platoons. Queue variance also increases (2.34 → 8.78), indicating better balancing of service with knowledge of time.

**w/o GAT:** this causes the worst degradation. ATT is up 25.3%, and AWT is up 38.8%. Even with perfect memory, uncoordinated agents fail. Non-stationarity comes back despite temporal modeling. Convergence also suffers, taking 1,800 episodes compared to 850 for the full model.

**w/o entropy:** ATT is only 2.8% higher, and AWT is only 3.5% higher; however, queue variance increases sharply by 570% (from 2.34 to 15.67). This variant attains its apparently good throughput by systematically starving side streets, making the fairness–efficiency trade-off explicit.

**w/o 3DQN:** ATT +6.3%, AWT +9.5%, time to convergence = 1,450 episodes. Contributions from both dueling DQN architecture and double Q-learning are substantial.

The full HC-STGRL algorithm is the only one to optimally trade off all criteria-maximum throughput performance with fairness. Figure 11 illustrates the ablation study.

**Figure 11:**
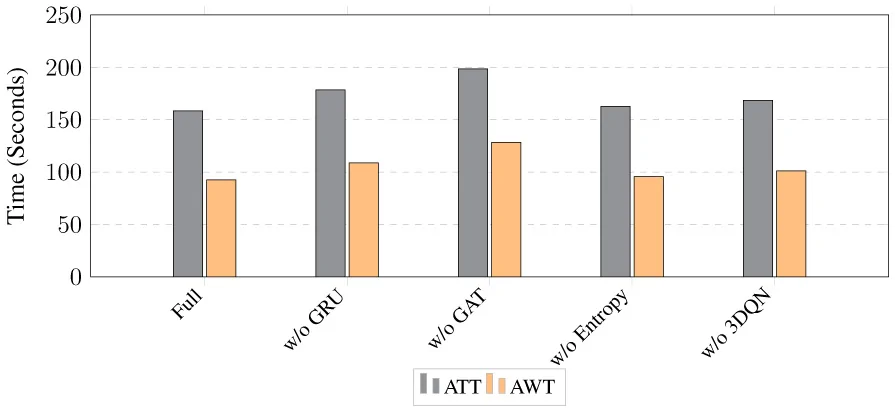
Ablation results. Removing GRU or GAT significantly hurts performance. Removing entropy hides unfairness behind seemingly good throughput. Full model balances all objectives.

To quantify the relative performance degradation when removing each component, we compute the percentage degradation Δ for each variant (Equation 42) relative to the full model:


$$\begin{array}{l} \Delta_{\text{ATT}}=\frac{{\text{ATT}}_{\text{variant}}-{\text{ATT}}_{\text{full}}}{{\text{ATT}}_{\text{full}}}\times 100\% \end{array}$$ (42)

The standard error of this degradation estimate is propagated from the individual standard deviations:


$$\begin{array}{l} \sigma_{\Delta }=\frac{1}{{\text{ATT}}_{\text{full}}}\sqrt{s_{\text{variant}}^{2}+{\left(\frac{{\text{ATT}}_{\text{variant}}}{{\text{ATT}}_{\text{full}}}\right)}^{2}s_{\text{full}}^{2}} \end{array}$$ (43)

Using this formulation, we find that removing the GAT causes the worst degradation: ATT increases by 25.3%±2.1% and AWT by 38.8% ± 2.5%. Even with perfect memory, uncoordinated agents fail. Non-stationarity returns despite temporal modeling. Convergence also suffers, taking 1,800 ± 78 episodes compared to 850 ± 45 episodes for the full model.

### 6.9 Training convergence analysis

Figure 12 shows training curves for HC-STGRL and key baselines. Several observations:

**Figure 12:**
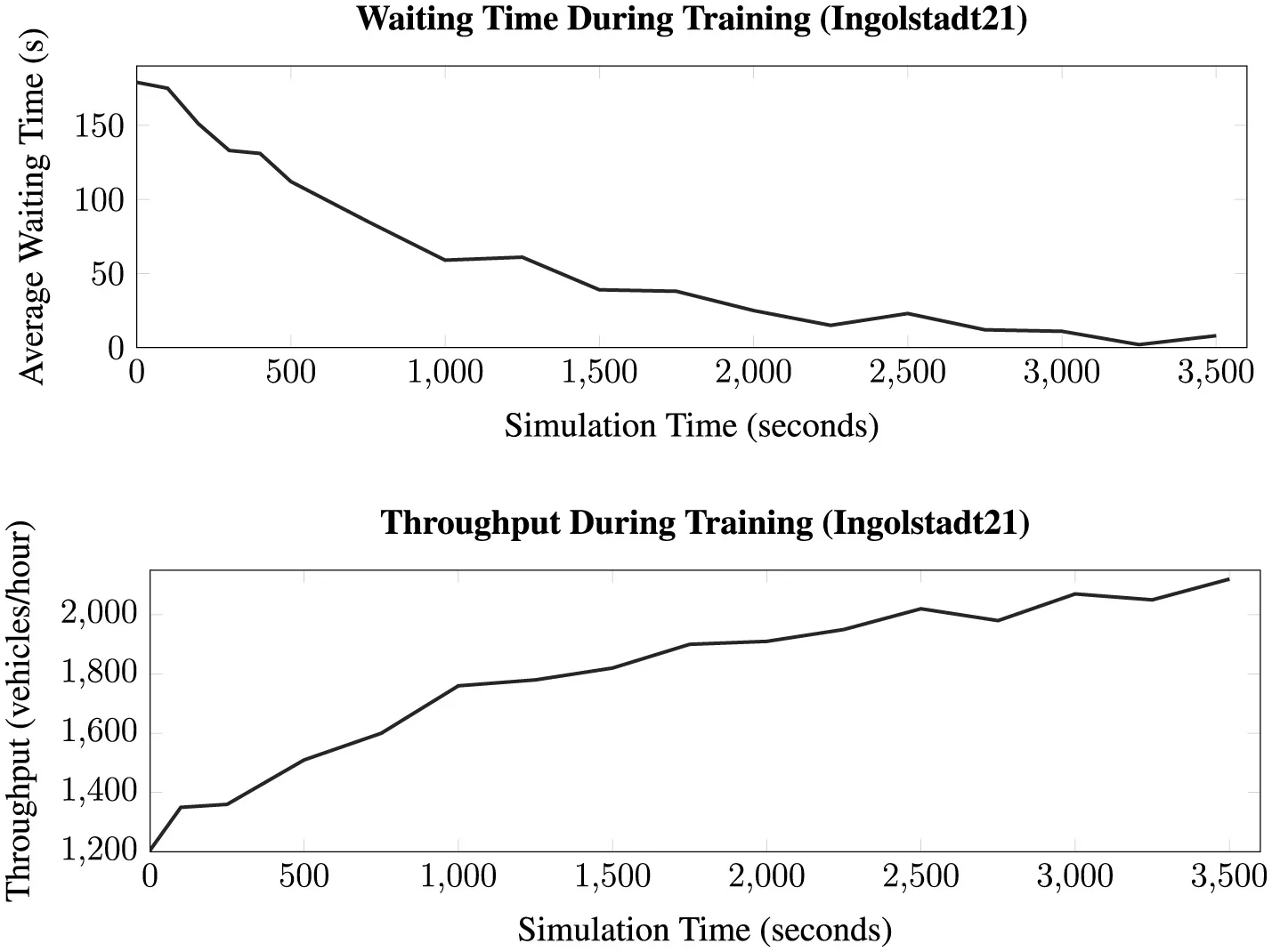
Training progress. Waiting time drops and throughput rises as GAT and GRU modules learn to coordinate. By 3,500 s, system stabilizes at low wait times and high throughput.

HC-STGRL exhibits the quickest convergence to the maximum reward value (850 episodes). The best baseline model, CoLight requires 1,250 episodes and settles at a lesser reward level. The variance of MPLight is very large; pressure rewards are apparently inconsistent. IDQN does not converge in any way, with rewards oscillating in the whole process. In our propose model two factors contribute to faster convergence: GRU allows richer representations of states, hence less exploration required; dueling architecture helps learn states faster irrespective of the action.

### 6.10 Computational complexity and deployment feasibility

We analyze the computational complexity, computational cost, memory consumption, communication overhead, scalability, and inference latency of HC-STGRL to assess its suitability for deployment in large-scale urban traffic networks.

Let *N* denote the number of intersections, *F* the input-feature dimension, *H* the GRU hidden dimension, and *K* the number of attention heads.

GRU local update for each agent: 𝒪(*H*^2^+*H*·*F*) GAT multi-head for each agent: $O\left(K\cdot |N_{i}|\cdot H^{2}\right)$

In road graphs, nodes are sparse. The maximum degree in an urban grid is either 4 or 5. This means that in our road graphs, |𝒩_*i*_| ≤ 5 for all nodes.

Total per-step computation: $O\left(N\cdot K\cdot |N_{\text{max}}|\cdot H^{2}\right)$, which is linear in *N* with small constant.

Since the maximum node degree remains bounded in urban road networks, the computational cost increases approximately linearly with the number of intersections rather than quadratically. Consequently, scaling from dozens to hundreds of intersections primarily increases runtime in proportion to the number of agents, supporting the suitability of HC-STGRL for large-scale urban traffic networks.

Inference takes milliseconds on embedded hardware, so HC-STGRL can run on existing traffic infrastructure without upgrades. The linear scaling in *N* means that even large networks such as Manhattan (196 intersections) update in under 13 ms on modest hardware, well within the typical 1–2 s control cycle.

Unlike centralized MARL approaches, whose joint state and action spaces often grow super-linearly with the number of agents, HC-STGRL performs only local GRU updates and neighborhood attention at each intersection. Therefore, the computational cost associated with scaling to larger road networks remains modest while preserving real-time inference capability.

#### 6.10.1 Memory and communication overhead

The decentralized, parameter-shared design keeps the memory footprint modest: each agent stores one GRU hidden state of dimension *H* and the shared GRU/GAT/dueling weights, so total parameter memory is independent of *N*, and per-step state memory grows only linearly as 𝒪(*N*·*H*). Communication is strictly local: at each control step an agent exchanges one *H*-dimensional embedding with each of its at most |𝒩_max_| neighbors, giving a per-step communication cost of 𝒪(*N*·|𝒩_max_|·*H*), again linear in the network size and requiring no central coordinator. This contrasts with centralized critics, whose communication and computation scale super-linearly with *N*.

#### 6.10.2 Scalability

Because computation, memory, and communication all scale linearly with *N*, while the node degree remains bounded in practical road networks, HC-STGRL maintains predictable computational cost as network size increases. The successful evaluation on the Manhattan network (196 intersections), together with the low inference latency reported in Table 14, supports the conclusion that the proposed framework remains suitable for large-scale real-time traffic signal control.

**Table 14:** Computational requirements and hardware feasibility.

Device	Peak performance	Inference time (196 nodes)	Deployment feasibility
ARM Cortex-A53 (typical traffic cabinet)	4.8 GFLOPS	12.3 ms	Yes
Intel Atom x5-Z8350 (industrial controller)	6.7 GFLOPS	8.7 ms	Yes
NVIDIA Jetson Nano (edge AI)	472 GFLOPS	0.8 ms	Yes
Cloud Server	Unlimited	less than 1 ms	Yes

### 6.11 Deployment considerations

While the evaluation is simulation-based, several practical factors govern real-world deployment. The sim-to-real gap is the foremost concern: although SUMO models car-following and lane-changing realistically (Section 5), unmodeled pedestrians, emergency vehicles, weather, and driver heterogeneity remain, and bridging this gap will require domain randomization during training and a supervised warm start from logged signal data before any live operation. **V2X and communication requirements** are modest by design, since only small embeddings are exchanged with immediate neighbors; nevertheless, a deployment must provide a low-latency local link (for example, fiber between adjacent cabinets or a dedicated short-range radio) and a graceful fallback to a fixed-time or max-pressure policy when links are lost. **Edge-computing integration** is well-matched to the linear-time, low-memory profile reported above, allowing inference to run inside the traffic cabinet and keeping raw sensor data local, which also benefits privacy (Chen et al., 2024). **Infrastructure constraints and sensor synchronization** must be respected: observations from neighboring intersections should be timestamped and aligned to a common control cycle, and clock drift across cabinets should be bounded so that exchanged embeddings refer to comparable intervals. Finally, **cybersecurity** is essential, because a controller that infers state from neighbors is exposed to spoofed or manipulated messages; authenticated inter-cabinet communication, anomaly detection on incoming embeddings, and the attention mechanism's ability to down-weight inconsistent neighbors together provide defense in depth, but a dedicated security analysis is beyond the present scope.

## 7 Limitations

Nevertheless, even with the strong points mentioned above, there are some limitations to this study which restrict the generality of the conclusions reached and the directions for future research.

- **Sim-to-real gap**. Although SUMO successfully simulates realistic vehicle behavior, there are many factors such as pedestrian traffic, emergency services, construction sites, weather conditions and delay in communication that remain untested until now. It would require validation on an actual testbed or high-fidelity digital twins before applying the algorithm in reality.
- **Communication reliance**. The GAT architecture assumes that there is reliable communication between vehicles. Whereas the robustness study takes into account potential failures of sensors, failure of the communication link, unlike those described in Section 6.5.1, might affect performance, and correlation or adversarial communication failures were not studied in detail.
- **Training data and compute requirements**. Converging requires hundreds of episodes and large amount of offline computations on a GPU. It is feasible to use it for pre-training offline but impossible for on-the-fly adaptation to rapidly changing networks.

## 8 Conclusion and future work

The HC-STGRL framework proposes a novel concept of traffic control by incorporating GRU-based temporal memory, multi-head GAT-based spatial attention, and a Dueling Double DQN learner into a fairness-aware multi-objective reward design. It solves two inherent problems of graph-based MARL controllers, namely, the absence of temporal anticipation capability and sensitivity to sensor faults by using two synthetic and two real-world networks. Compared to the best baseline, CoLight, our proposed model HC-STGRL provides up to 11.3% reduction in travel time and up to 14.9% reduction in waiting time in real-world networks where the gap increases with network complexity. Under the sensor failure rate of 20%, HC-STGRL shows only a performance degradation of 25.4%, while CoLight and MaxPressure degrade by 44.7 and 62.4%, respectively. The spatial entropy component decreases the variance in queue length approximately by an order of magnitude compared to the version.

These results are promising, but several areas remain for future exploration. Extending the framework to multi-modal traffic that includes pedestrians makes the system more inclusive and aligned with modern urban mobility goals. Federated learning presents an opportunity to train models across multiple cities without sharing sensitive traffic data, leveraging diverse traffic patterns for improved generalization while addressing privacy concerns. Moreover, the empirical comparison with recent transformer-based traffic signal controllers is currently qualitative and should be extended to include a comprehensive quantitative evaluation. By uniting spatial coordination with temporal anticipation in a single deployable controller, HC-STGRL advances adaptive traffic signal control beyond the static-snapshot paradigm. We believe that the framework provides a solid foundation for subsequent work on robust, fair, and explainable intelligent transportation systems.

## Figures and Tables

**Table 15:** List of abbreviations.

Abbreviation	Definition
3DQN	Dueling double deep Q-Network
ATT	Average travel time
AWT	Average wait time
DQN	Deep Q-Network
DDQN	Double deep Q-Network
GAT	Graph Attention Network
GNN	Graph neural network
GRU	Gated recurrent unit
GWE	Green Wave Efficiency
HC-STGRL	Hybrid coordinated spatio-temporal graph reinforcement learning
IDQN	Independent deep Q-Network
ITS	Intelligent transportation systems
LSTM	Long short-term memory
LWR	Lighthill–Whitham–Richards (traffic flow model)
MARL	Multi-agent reinforcement learning
MDP	Markov decision process
MAMDP	Multi-agent Markov decision process
MLP	Multi-layer perceptron
RL	Reinforcement learning
RNN	Recurrent neural network
SUMO	Simulation of urban MObility
TD	Temporal difference
TSC	Traffic signal control
V2X	Vehicle-to-everything (communication)

**Table 16:** List of principal symbols.

Symbol	Meaning
𝒢 = (𝒱, ℰ)	Road network graph: intersections 𝒱 and roads ℰ
𝒩_*i*_	Set of neighboring intersections of agent *i*
$o_{i}^{t}$	Local observation of agent *i* at time *t*
$q_{i}^{t},d_{i}^{t},w_{i}^{t}$	Queue lengths, lane densities, waiting times at agent *i*
$p_{i}^{t},\Phi_{i}^{t}$	Max-pressure and current-phase encoding at agent *i*
$h_{i}^{t}$	GRU hidden (temporal memory) state of agent *i*
$z_{i}^{t},r_{i}^{t},{\tilde{h}}_{i}^{t}$	GRU update gate, reset gate, candidate state
*e*_*ij*_, α_*ij*_	Unnormalized and normalized GAT attention coefficients
W_gat_, a	GAT shared projection matrix and attention vector
*K*	Number of GAT attention heads
$X_{i}^{t}$	Spatio-temporal state representation of agent *i*
$R_{i}^{t}$	Reward of agent *i* at time *t*
ℋ(·)	Shannon entropy of the per-lane queue distribution
ω_1_, …, ω_4_	Reward-term weighting coefficients
*V*(·), *A*(·, ·), *Q*(·, ·)	State-value, advantage, and action-value functions
θ, θ^−^	Online and target network parameters
γ, α, ϵ	Discount factor, learning rate, exploration rate
𝒟	Experience replay buffer
τ_yellow_	Yellow clearance duration
Statistical symbols (Section 5.6)	
*R*	Number of independent runs (5 in our experiments)
*M*_*r*_	Metric value obtained in run *r* (e.g., ATT, AWT)
$\bar{M}$	Sample mean across *R* runs
*s*_*M*_	Sample standard deviation across *R* runs
CI_95%_	95% confidence interval for the true mean
*t*_*R*−1, 0.975_	Critical value of Student's *t*-distribution
*d*	Cohen's *d* effect size
*s*_pooled_	Pooled standard deviation
Δ	Percentage degradation when removing a component
ℒ(*f*)	Performance loss at sensor failure rate *f*
ℐ	Relative improvement over a baseline

## Data Availability

The original contributions presented in the study are included in the article/supplementary material, further inquiries can be directed to the corresponding author.

## Nomenclature

For clarity and ease of reference, the abbreviations and principal mathematical symbols used throughout this manuscript are summarized in Tables 15, 16, respectively.
